# Antineoplastic Activity of Podophyllotoxin and Juniper Extracts Encapsulated in MPEG-*b-*PLA Diblock Copolymer Micelles in Cutaneous Squamous Carcinoma Cells

**DOI:** 10.3390/ijms26115167

**Published:** 2025-05-28

**Authors:** Radostina G. Kalinova, Ivaylo V. Dimitrov, Yana Ilieva, Dimitar B. Iliev, George A. Miloshev, Dessislava N. Staneva, Maya M. Zaharieva, Aleksandrina Nesheva, Galya Staneva, Diana I. Ivanova, George Angelov, Hristo M. Najdenski

**Affiliations:** 1 Institute of Polymers, Bulgarian Academy of Sciences, 1113 Sofia, Bulgaria; kalinova@polymer.bas.bg (R.G.K.); dimitrov@polymer.bas.bg (I.V.D.); 2 National Centre of Excellence “Mechatronics and Clean Technologies”, 8 Kliment Ohridski Blvd, 1756 Sofia, Bulgaria; 3 The Stephan Angeloff Institute of Microbiology, Bulgarian Academy of Sciences, 1113 Sofia, Bulgaria; illievayana@gmail.com (Y.I.); zaharieva26@yahoo.com (M.M.Z.); hnajdenski@abv.bg (H.M.N.); 4 The Rumen Tsanev Institute of Molecular Biology, Bulgarian Academy of Sciences, 1113 Sofia, Bulgaria; diliev@bio21.bas.bg (D.B.I.); karamolbiol@gmail.com (G.A.M.); dessysta@gmail.com (D.N.S.); 5 Institute of Biophysics and Biomedical Engineering, Bulgarian Academy of Sciences, 1113 Sofia, Bulgaria; nescheva@gmail.com (A.N.); gstaneva@bio21.bas.bg (G.S.); 6 Institute of Chemical Engineering, Bulgarian Academy of Sciences, 1113 Sofia, Bulgaria; georgeangelov@yahoo.com

**Keywords:** antineoplastic activity, apoptosis, caspases, drug nanocarriers, epidermoid carcinoma, FACS, comet assay, *Juniperus* L., podophyllotoxin, Laurdan generalized polarization

## Abstract

Nanotechnology offers alternative approaches to the discovery of anticancer drugs. Hydrophobic bioactive components can be included in the cores of amphiphilic nanocarriers, which leads to the formation of a water-dispersible product with improved bioavailability, facilitated excretion, and reduced systemic toxicity in the treated organisms. This study was aimed at the formation of polymer nanocarriers, loaded with anticancer drug precursor podophylotoxin (PPT) or PPT-containing juniper leaf extracts, seeking to study their antineoplastic activity in A-431 epidermoid carcinoma cells and HaCaT normal keratinocytes. The amphiphilic, biodegradable, and biocompatible MPEG-*b*-PLA diblock copolymer was self-assembled in aqueous media into nanosized particles, whose physicochemical characteristics were studied by dynamic light scattering, transmission electron microscopy, and other methods. High encapsulation efficiency was determined for the PPT component-loaded micelles. DNA fragmentation, cell cycle arrest, nuclear condensation, membrane lipid order assessment, reactive oxygen species, and apoptosis induction by the loaded nanocarriers in A-431 or HaCaT cells were analyzed by the comet assay, FACS, Hoechst DNA staining, Laurdan generalized polarization, and other methods. As a result of various cellular processes induced by the PPT component-loaded nanoparticles, effector caspase-3 and caspase-7 activation showed selectivity towards tumor cells compared to the normal cells. The newly obtained PPT-containing nanoparticles have applications as potential drugs in the prospective nanomedicine.

## 1. Introduction

Cancer is the second-leading cause of human mortality in the world after cardiovascular diseases. Many natural compounds have effective anticancer properties [1]; however, their clinical application is hampered by their low water solubility, poor bioavailability, and high toxicity in the treated organisms. Despite the huge progress in the treatment of cancer, high efficiency and reduced toxicity are still required in the development of new drugs.

**Figure 1:**
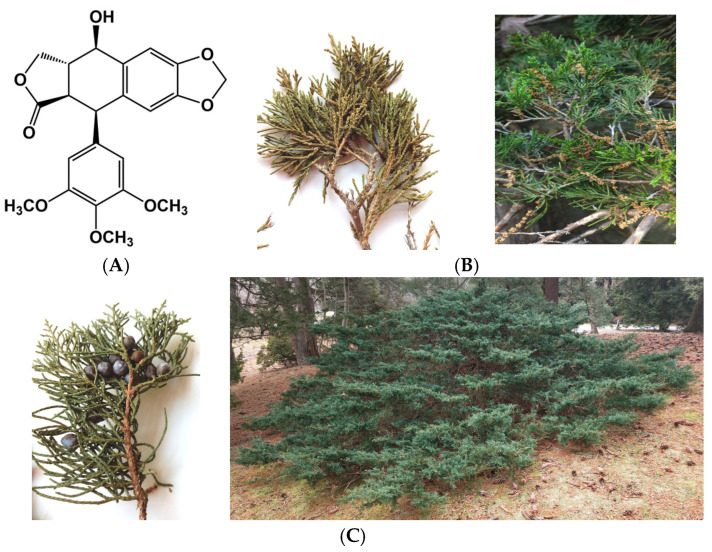
Structure of podophyllotoxin (**A**). Specimens and plant representatives of *Juniperus horizontalis* (**B**) and *Juniperus virginiana* ‘Grey Owl’ (**C**), containing podophyllotoxin in their leaves.

Podophyllotoxin (PPT, Figure 1A) is a highly efficient natural anticancer compound [2]. Podophyllin is a resin, containing PPT, from the roots of *Podophyllum peltatum* L., which is applied in dermatology (for fungal infections, genital warts, and benign, premalignant, and malignant dermatoses), but it exhibits many cutaneous and systemic side effects [3]. PPT is obtained industrially from the rhizomes of *Podophyllum peltatum* L. (American Mayapple) or *Sinopodophyllum hexandrum* (Royle) T. S. Ying (Indian Mayapple). However, these plants have become endangered species because of their intensive industrial exploitation.

In the search for other natural sources of PPT, some juniper species were identified to contain PPT and other lignans in their leaf extracts, which exhibited efficient antiproliferative properties. The PPT content in the leaves of junipers, as evergreen plant species, was found to be stable throughout the year. Junipers, growing as shrubs, were considered more suitable for industrial cultivation than junipers growing as trees. For this reason, representatives of *Juniperus horizontalis* Moench (creeping juniper, Figure 1B) and the *Juniperus virginiana* ‘Grey Owl’ cultivar (Figure 1C), growing like shrubs, were selected as research objects for the present study. The leaf extracts of *J. horizontalis* (JHE) and ‘Grey Owl’ juniper (GO) were found to contain PPT in the range of 0.38–0.39% [4].

The cellular mechanisms of the anticancer activity of PPT include binding to tubulin [5], which interrupts the assembly and disassembly of the microtubules during mitosis, leading to DNA damage and G2/M cell cycle arrest [6]. In this process, the activation of proapoptotic proteins p21, p27, DR4, DR5, Bad, Bax, Apaf-1, etc., leads to cytochrome c release in the cytosol [7], activation of caspases (initiator caspases-8/-9 and executor caspase-3) by the extrinsic (death receptor) and/or intrinsic (mitochondrial) apoptotic pathways [8].

In the search for compounds with increased anticancer activity and reduced toxicity, plenty of PPT derivatives have been synthesized [9]. As a result, highly efficient anticancer drugs such as etoposide, teniposide, and etopophos have been discovered for the treatment of various forms of cancer via structural modifications of PPT (glycosylation of the hydroxyl group in the C-ring of the molecule) [10]. These PPT derivatives bind to DNA and are topoisomerase II inhibitors; however, their medicinal application is limited by their various side effects, as well as poor bioavailability and impaired hematopoiesis [11].

Nanotechnology offers different approaches to the discovery of new anticancer drugs [12]. Hydrophobic substances can be included in the cores of amphiphilic nanocarriers, which leads to water-dispersible nanomaterials, aimed at improved bioavailability, facilitated excretion, and reduced systemic toxicity in the treated organisms [13,14]. Various phytocompounds have been included in different nanoparticle-based systems for the effective treatment of cancer [15]. In this context, water-dispersible podophyllotoxin–polyacrylic acid conjugate micelles (particle size of about 215 nm) were obtained, and their improved cytotoxicity in MCF-7 and MDA MB-231 breast cancer cells in comparison with PPT was determined [16]. In other study, conjugates of PPT and poly(*L-*glutamic acid)-*g*-methoxy poly(ethylene glycol) (PLG-*g*-mPEG) via ester bonds were assembled into nanoparticles (size of about 100 nm), which showed an increased maximum tolerated dose (MTD) and enhanced antitumor activity in comparison with free PPT in MCF-7/ADR xenograft tumors [17]. PPT-conjugated stearic acid-grafted chitosan oligosaccharide micelles (PPT-CSO-SA) were characterized as potential anti-glioma nanodrugs. The antiproliferative activity of PPT-CSO-SA micelles was found to be stronger than that of PPT in the induction of G2/M cell cycle arrest and apoptosis of glioma cells [18]. A PPT-containing nanomaterial, namely a peptide–polyethylene glycol (PEG)–podophyllotoxin (PEG-Pep-PODO) conjugate, included a short peptide as a ligand of E-selectin. As targeted protein for nanodrug discovery, E-selectin was found to be involved in tumor growth and was used as a marker for the metastatic potential of some cancers (colorectal cancer, etc.). The obtained PPT conjugate was a water-dispersible product that showed decreased systemic toxicity and improved pharmacokinetics in MCF-7 (estrogen receptor-positive) breast cancer xenograft tumors in comparison with free PPT [19].

The present study is the first investigation of the antineoplastic activity of podophyllotoxin or PPT-containing juniper extracts encapsulated in biodegradable and biocompatible MPEG-*b-*PLA diblock copolymer micelles as potential nanodrugs for the treatment of cutaneous squamous cell carcinoma (CSCC, epidermoid carcinoma).

PPT or juniper extracts were encapsulated in poly(ethylene glycol)-*b-*poly(*D,L-*lactide) (MPEG-*b*-PLA)-based micelles and their physicochemical characteristics were determined. The effective antiproliferative activity of the obtained micelles loaded with bioactive components was determined after the treatment of A-431 epidermoid carcinoma cells and HaCaT normal keratinocyte cells. The induction of DNA fragmentation, nuclear condensation and shrinkage, cellular membrane lipid-ordering effects, and reactive oxygen species (ROS) induction followed by cell cycle arrest and apoptosis in the studied cell lines treated with PPT-containing nanocarriers were analyzed via comet assay, Hoechst DNA staining, Laurdan generalized polarization (GP) measurements, FACS, and other methods. As a result of the various cellular processes induced by the PPT component-loaded nanoparticles, effector caspase-3 and caspase-7 activation showed selectivity towards tumor cells compared to normal cells.

Further preclinical studies should be performed to reveal the expected decreased toxicity of the PPT-containing polymer micelles in in vivo conditions due to their expected increased bioavailability in the blood and facilitated excretion from the treated organisms.

## 2. Results and Discussion

### 2.1. Preparation, Drug Loading, and Physicochemical Characterization of Micelles

#### 2.1.1. Copolymer Synthesis, Preparation, and Drug Loading of Micelles

**Figure 2:**
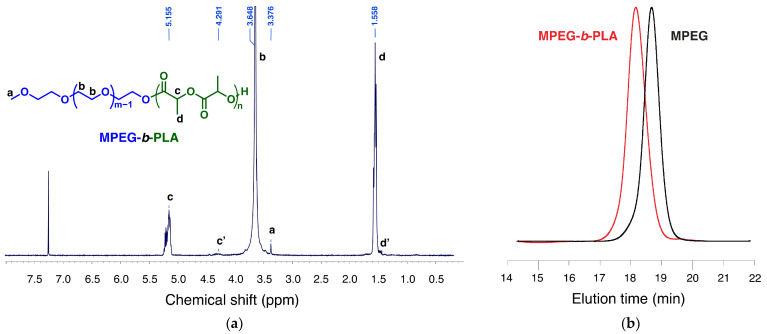
MPEG-*b*-PLA block copolymer characterization via ^1^H-NMR (**a**) and size exclusion chromatography in tetrahydrofuran vs. polystyrene narrow molar mass standards (**b**). Abbreviation: MPEG-*b*-PLA—methoxy poly(ethylene glycol)-*b-*poly(*D,L-*lactide).

The biodegradable and biocompatible MPEG-*b*-PLA block copolymer was synthesized via ring-opening polymerization of a *D,L*-lactide monomer initiated by an MPEG macroinitiator, applying an already described procedure [20]. The polymerization was performed in bulk at a temperature of 125 °C. The purified block copolymer was analyzed by ^1^H-NMR spectroscopy and size exclusion chromatography (SEC). The degree of polymerization of the lactide monomer (DP_n_ = 30) was calculated from the ratio of the integral intensities of oxyethylene protons from the macroinitiator at 3.65 ppm and the methine protons of the LA-repeating units of the polyester block at 5.16 ppm (Figure 2a). The obtained polymer was characterized by narrow dispersity (Đ = 1.11) and a monomodal molar mass distribution, as evident from the size exclusion chromatography analyses (Figure 2b).

The synthesized and characterized well-defined MPEG-*b*-PLA block copolymer was further used for the preparation of nanoparticles designed for the delivery of poorly water-soluble anticancer substances. The drug-loaded nanoparticles were successfully prepared by applying a two-step procedure. Firstly, the solvent evaporation method was used to obtain MPEG-*b*-PLA copolymeric micelles. Afterwards, an ethanolic solution of the corresponding bioactive substance was injected dropwise into aqueous dispersions of preformed micelles under continuous stirring. Finally, the ethanol was evaporated, and the concentration of the micellar dispersions was adjusted to 1 mg mL^−1^. The encapsulation efficiency (EE) and loading capacity of (LC) of MPEG-*b*-PLA micelles were calculated for PPT and two juniper extracts (JHE and GO) according to Equations (1) and (2) (Section 3.5) and are summarized in Table 1. As is evident, the encapsulation efficiency values for the MPEG-*b*-PLA micelles varied distinctly for the different active substances. The highest EE of about 98 wt% was obtained for PPT-loaded micelles. The juniper extract-loaded micelles were characterized by lower EE of 74 wt% for JHE and 83 wt% for GO extracts. The calculated LC values were 9%, 7.4%, and 8.3%, for PPT, JHE, and GO, respectively.

#### 2.1.2. Physicochemical Characterization of Micelles

**Figure 3:**
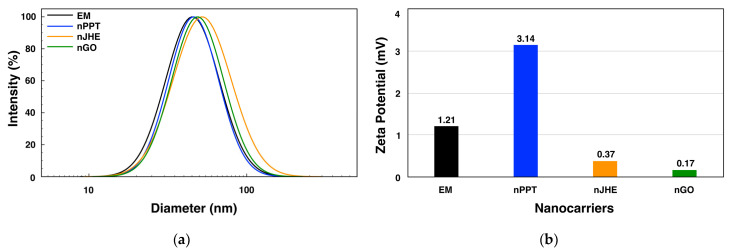
Size distributions (**a**) and zeta potentials (**b**) obtained from dynamic light scattering analyses of empty micelles (EM: d = 45 nm, PdI 0.169, ζ = 1.21 mV) and PPT-loaded (nPPT: d = 46 nm, PdI 0.145, ζ = 3.14 mV), JHE-loaded (nJHE: d = 52 nm, PdI 0.205, ζ = 0.37 mV), and GO-loaded (nGO: d = 49 nm, PdI 0.166, ζ = 0.17 mV) block copolymer micelles.

The particle size, polydispersity index, and surface charge of a nanocarrier are known to play an important role in drug release, the circulation time, interactions with biological membranes, the absorption rate, and its biodistribution within specific organs [21]. Therefore, the size distribution and zeta potentials of both empty and bioactive substance-loaded micelles were measured using DLS, and the data are presented in Figure 3 and Table 1.

The results showed that the empty MPEG-*b*-PLA micelles had an average particle diameter of 45 nm and a narrow size distribution (PdI = 0.169). After loading with the active substances, the average diameters of the MPEG-*b*-PLA NPs slightly increased to 46.00, 51.63, and 48.68 nm for the PPT-, JHE-, and GO-loaded micelles, respectively. This could be attributed to the micelles’ core expansion as a result of the relatively high drug loading of the nanoparticles [22,23]. The loaded micelles also showed monomodal and relatively narrow size distributions, with PdI values in the 0.15–0.2 range.

Furthermore, the zeta potentials of the empty and loaded MPEG-*b*-PLA micelles were measured. In both cases, the micelles were found to be slightly positively charged, with zeta potential values in the range of 0.17 to 3.14 mV (Figure 3b and Table 1). This value of the surface charge, close to 0 mV for all particles, revealed that the PEG corona effectively shielded the PLA core and indicated that the active substances were located mainly in the particles’ cores.

**Figure 4:**
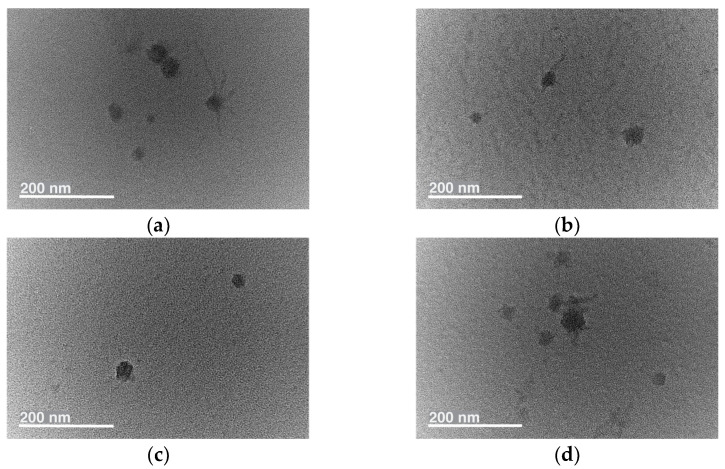
Transmission electron microscopy images of empty (**a**), PPT-loaded (**b**), JHE-loaded (**c**), and GO-loaded (**d**) block copolymer (MPEG-*b*-PLA) micelles.

The morphologies (size and shape) of the nanoparticles were determined through transmission electron microscopy (TEM) (Figure 4). The obtained results indicated that all nanoparticles had a spherical morphology, with average diameters close to those obtained by the dynamic light scattering (DLS) measurements.

#### 2.1.3. In Vitro Drug Release Evaluation

**Figure 5:**
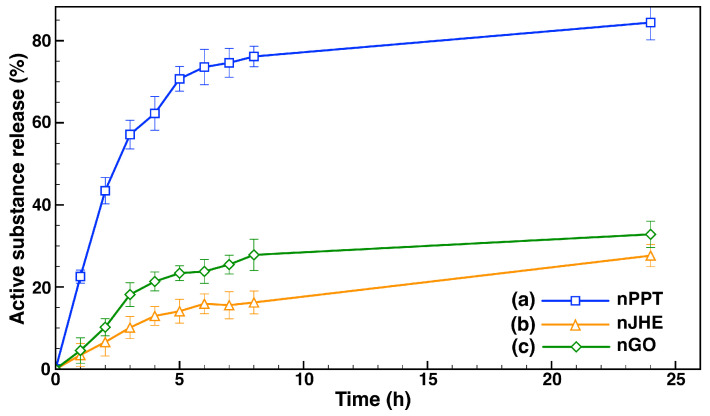
In vitro release profiles of active substances in phosphate buffer (PBS, pH 7.4) containing 10% (*v/v*) ethanol from (**a**) PPT-loaded (nPPT), (**b**) JHE-loaded (nJHE), and (**c**) GO-loaded (nGO) nanocarriers. The data are expressed as the mean value ± SD, *n* = 3.

The release of the three different active compounds from the block copolymer micelles was followed and evaluated in phosphate buffer (PBS, pH 7.4). The release media comprised 10% (*v*/*v*) ethanol. The amounts of released active substances from the respective micellar carriers at predetermined time intervals were quantified via UV/Vis spectroscopy. The cumulative percentages of drug release as a function of time are presented in Figure 5.

All three profiles obtained for the different micellar formulations (nPPT, nJHT, and nGO) showed a burst in drug release during the first 6–8 h of evaluation, followed by much slower release during the next few hours. The initial burst release followed first-order-like kinetics and could be attributed to the release of the active substances, which were located on the peripheries of the micelles’ cores, close to the hydrophilic shell. The second stage of slow drug release was due to the release of active compounds from the inside of the micelle’s core. However, there was a significant difference in the amount of the released PPT for both extracts from the copolymer micelles. After the first 6 h of evaluation, 74% of the PPT had migrated through the membrane into the release media. At the 24th hour, more than 80% of the PPT was detected in the release media. In contrast, only small amounts of the extracts were released from the copolymer micelles. During the first eight hours of evaluation, just 16% of JHE and 24% of the GO extracts were released from the micelles, followed by slower drug release, reaching 27% and 33% after 24 h of evaluation. The much slower release of the JHE and GO extracts could be attributed to their complex compositions, in which PPT was just one of many other components. This, most likely, led to stronger hydrophobic interactions between the extracts’ components and the micelles’ core-forming macromolecules.

#### 2.1.4. Stability Test

**Figure 6:**
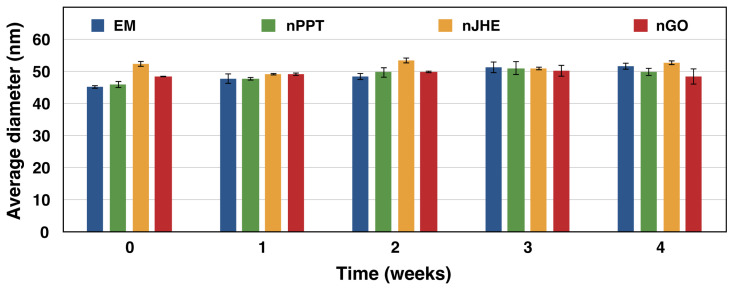
Stability of copolymer micelles, i.e., empty micelles (EM), PPT-loaded (nPPT), JHE-loaded (nJHE), and GO-loaded (nGO) nanosized micelles, assessed by dynamic light scattering measurements in aqueous media after various storage intervals at 4 °C.

The physical stability of empty and loaded MPEG-*b*-PLA micelles at 4 °C was assessed by DLS measurements (Figure 6). As is evident from the obtained results, the micellar formulations did not show any noticeable change in particle size or size distribution over a period of 30 days. This is an indication that both the empty and bioactive component-loaded micelles were stable for at least 1 month. The micelles’ enhanced stability is attributed to the presence of a nonionic hydrophilic MPEG shell.

### 2.2. Mechanism of Antineoplastic Activity of Nanocarriers Loaded with Podophyllotoxin or Juniper Leaf Extracts

#### 2.2.1. Cytotoxic Effects of PPT-Containing Bioactive Components Loaded in Nanocarriers

**Figure 7:**
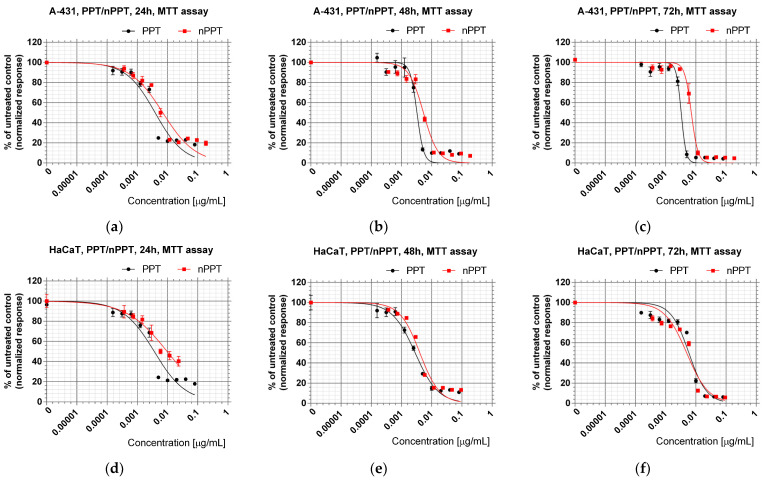
Sigmoidal graphs derived from MTT assay after treatment with podophyllotoxin (PPT) or PPT-loaded nanosized micelles (nPPT) in A-431 cell line for 24 (**a**), 48 (**b**), and 72 (**c**) hours and in HaCaT cell line for 24 (**d**), 48 (**e**), and 72 (**f**) hours.

The cytotoxic activity of the obtained PPT component-loaded nanoparticles in comparison with the individual bioactive ingredients was determined using A-431 epidermoid carcinoma and HaCaT spontaneously immortalized non-tumorigenic keratinocyte cells. The concentration-dependent antiproliferative effects of the treatment of the studied cell lines with the corresponding bioactive components encapsulated in nanomicelles, in comparison with the individual reagents, are shown in Figure 7.

*Juniperus horizontalis* leaf extract (JHE) showed higher antiproliferative activity than Grey Owl juniper leaf extract (GO) in A-431 cells (preliminary experiments); thus, only JHE was chosen for further in-depth bioactivity analyses. The antiproliferative properties of the nanocarriers loaded with bioactive components—podophyllotoxin (nPPT) or PPT-containing juniper leaf extract (nJHE)—and the corresponding free bioactive components—podophyllotoxin (PPT) and juniper leaf extract (JHE)—were evaluated via their IC_50_ values in treated tumor and normal cells (Table 2). The corresponding IC_50_ values were calculated as half-maximum growth-inhibitory concentrations derived from the dose–response curves of the MTT tests (Figure 7). Lower IC_50_ values indicated the higher activity of the tested sample.

The cutaneous side effects of PPT have been described in detail in the literature [3] i.e., it is also cytotoxic towards normal, non-tumorigenic skin cells. Our results (Table 2) were in line with this observation. Besides the expected high cytotoxicity of PPT, nPPT was also cytotoxic at very low concentrations (nanograms per mL). The results showed that the PPT-containing agents exhibited higher cytotoxicity after 48 h of treatment (Table 2, Figure 7). The IC_50_ at 48 h for PPT-containing samples (PPT and nPPT) varied from 0.003 to 0.005 µg/mL PPT content. The IC_50_ at 48 h for JHE-containing agents (JHE and nJHE) was in the range of 0.16 to 0.7 µg/mL JHE content. 

In summary, the results showed that the polymer micelles (nPPT, nJHE), loaded with PPT and PPT-containing JHE, retained the high antiproliferative activity of the starting hydrophobic bioactive components. However, future preclinical studies should be performed to reveal the expected decreased toxicity of the PPT-containing polymer micelles in in vivo conditions due to their increased bioavailability and facilitated excretion from the treated organisms.

Specific concentrations of the loaded nanocarriers—nPPT at 0.005 µg/mL and nJHE at 0.4 µg/mL—were chosen for the treatment of the tumor cell line A-431 for the next in-depth analysis of caspase activation, reactive oxygen species induction, etc., in order to determine the effects of this possible therapeutic concentration on the tumor cell line and on normal cells. These concentrations were within the ranges of the IC_50_ values of nPPT and nJHE after the treatment of A-431 cells for 48 h (Table 2).

The results of the MTT test confirmed that the MPEG-*b*-PLA-based empty micelles (EM) demonstrated low cytotoxicity in A-431 epidermoid carcinoma or HaCaT normal cells. The IC_50_ values of the MPEG-*b*-PLA-based nanocarrier (empty micelles, EM) were found to be above the highest concentration that was reasonable to test in a 96-well plate (40 µg/mL). Therefore, the cytotoxicity of the EM in the studied cancer and normal cells was estimated by the determination of the percentage of viable cells observed after the treatment of the corresponding cell lines with different concentrations of unloaded micelles (Table 3). At the IC_50_ at 48 h in nJHE (0.4 µg/mL JHE content), the content of the nanocarrier was 5.3 µg/mL. At this amount of empty nanocarrier, the cell viability of the two lines was approximately 83% after 48 h of treatment, meaning that the micelles exhibited very low cytotoxicity. At 0.005 µg/mL of PPT content, the amount of the micelles in nPPT was 0.06 µg/mL, making the cytotoxic effect of empty micelles negligible.

#### 2.2.2. Activation of Caspase-3 and Caspase-7 as a Sign of Apoptosis Induction in A-431 and HaCaT Cells

**Figure 8:**
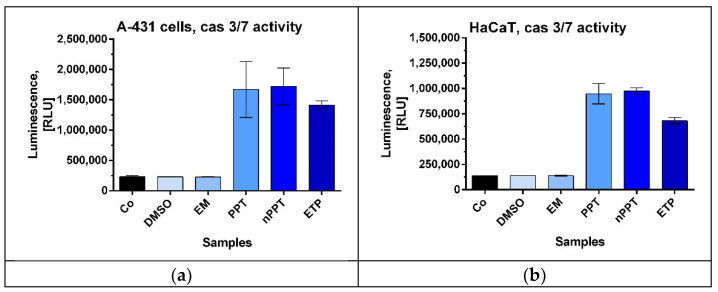
Caspase-3 and caspase-7 activity in A-431 (**a**) and HaCaT (**b**) cell lines treated with podophyllotoxin-containing agents for 24 h. The concentrations of PPT and the PPT-loaded nanocarrier were 0.005 µg/mL, and the concentration of etoposide was 50 µM. Abbreviations: RLU—relative luminescence units; PPT—podophyllotoxin; nPPT—PPT-loaded nanosized micelles; ETP—etoposide; DMSO—dimethyl sulfoxide, vehicle; EM—empty micelles; Co—control, untreated cells.

Caspase-3 and caspase-7 are both executioner caspases that are activated by the initiator caspases of both the extrinsic and intrinsic pathways. These executioner (effector) caspases cause the well-known chromatin and nuclear condensation and ultimately lead to the degradation of the cell during apoptosis [24]. The analysis of the total caspase-3 and caspase-7 activity of the treated cells showed that the studied bioactive agents significantly increased their activity in comparison with the control and the vehicle-treated samples (Figure 8). Of major significance, the induction was greater than that of the applied concentration of etoposide, a compound known to induce apoptosis. Moreover, the induction was greater (by more than 50%) in the tumor cell line in comparison with the normal keratinocytes. nPPT had slightly greater average activity than PPT. The EM did not increase the caspase activity. Therefore, the nanocarrier loaded with PPT induced caspase-3 and caspase-7 activity and hence induced apoptosis in epidermoid carcinoma A-431 cells. It demonstrated good selectivity in terms of being weaker in inducing caspase-3 and caspase-7 activity in the HaCaT normal cell line.

#### 2.2.3. Hoechst Assay for DNA Staining, Showing Apoptosis Induction in A-431 and HaCaT Cells Treated with PPT-Containing Bioactive Components

The A-431 and HaCaT cells were treated with the individual bioactive components PPT and JHE, or with the corresponding loaded nanocarriers. Then, a blue fluorescent dye, Hoechst 33342, was used to visualize DNA during the middle stage of apoptosis in the treated cell lines. The vehicle dimethyl sulfoxide (DMSO) and the EM did not induce any effects. In contrast, clear time- and concentration-dependent chromatin and nuclear condensation and shrinkage was observed to be induced by the PPT-containing extracts and samples, which is a hallmark of apoptosis. The results showed that the PPT and PPT-containing JHE extract-loaded MPEG-*b*-PLA micelles induced efficient apoptosis in A-431 and HaCaT cells (Table 4).

#### 2.2.4. Rise in Intracellular Reactive Oxygen Species (ROS) Induced by PPT-Containing Bioactive Components

**Figure 9:**
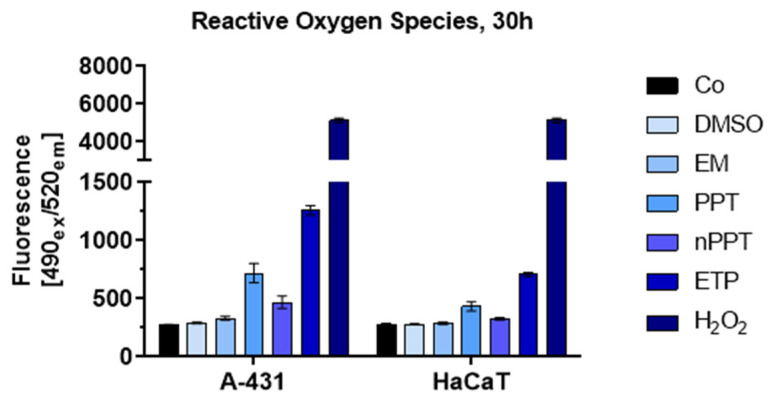
Reactive oxygen species (ROS) generation by the studied PPT agents after the treatment of A-431 and HaCaT cell lines for 30 h. Hydrogen peroxide (H_2_O_2_) (0.35%) was used as a positive control. PPT and nPPT were used at a 0.005 µg/mL concentration of the active ingredient, and etoposide was used at a concentration of 50 µM. Abbreviations: PPT—podophyllotoxin; nPPT—PPT-loaded nanosized micelles; ETP—etoposide; DMSO—dimethyl sulfoxide, vehicle; EM—empty micelles; Co—control, untreated cells.

Besides the positive control, hydrogen peroxide (H_2_O_2_) at 0.35%, etoposide at 50 µM was the agent that induced the greatest amount of ROS. Moreover, the values associated with PPT always exhibited a significant difference compared to the values of the control. Notably, in the tumor A-431 cell line, there was a significant difference in favor of nPPT (which induced ROS to a significantly greater extent compared to the control), while, in the normal HaCaT keratinocytes, there was no significant difference compared to the control (Figure 9). Therefore, nPPT again demonstrated good selectivity, i.e., it induced a greater amount of ROS in the tumor cell line compared to the normal one. The EM only slightly induced ROS compared to the control in the tumor cells and to an even smaller extent in the non-tumorigenic cells.

#### 2.2.5. Membrane Lipid Order Assessment of PPT Versus nPPT in A-431 and HaCaT Cells

The membrane lipid order assessment showed the opposite effects of PPT versus nPPT and different effect patterns in the two cell lines. The plasma membrane is the first cellular structure encountered by therapeutic agents, and its structural order plays a crucial role in determining drug uptake, distribution, and the cellular response. Laurdan generalized polarization (GP) is a fluorescence-based parameter that reflects lipid packing and the polarity of the membrane environment. Higher GP values indicate reduced water penetration and increased lipid order, typically associated with the liquid-ordered (Lo) phase found in cholesterol-rich membranes. Conversely, lower GP values correspond to increased membrane fluidity and disorder, characteristic of the liquid-disordered (Ld) phase. Thus, Laurdan GP serves as a sensitive tool for the assessment of changes in membrane biophysical states in response to cellular perturbations or treatments.

**Figure 10:**
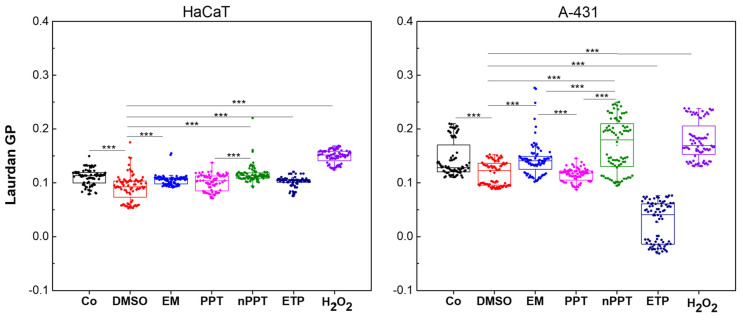
Laurdan generalized polarization (GP) measurements in HaCaT and A-431 cells before treatment (Co, black points) and after treatment. Cells were treated (24 h incubation) with DMSO (red), an empty nanocarrier (EM, blue), podophyllotoxin (PPT, magenta), nanoencapsulated podophyllotoxin (nPPT, green), etoposide (ETP, navy), or hydrogen peroxide (H₂O₂, purple). The points represent four replicates, with each replicate measured 20 times; box plots show the means with SD values. Statistical comparisons between treatment groups were performed using one-way ANOVA followed by Tukey’s post hoc test. *** denotes *p* < 0.001.

In this study, the baseline GP values were higher in A-431 cells compared to HaCaT cells, indicating that the cancerous A-431 cell line possesses more ordered membranes under control conditions (Figure 10). This intrinsic difference suggests that cancer cell membranes may be more structured, potentially due to increased cholesterol content. Such structural differences could affect both drug permeability and membrane-mediated signaling.

Upon treatment with DMSO, both cell lines exhibited a reduction in GP, but the effect was more pronounced in A-431 cells, suggesting the enhanced susceptibility of cancer cell membranes to solvent-induced fluidization. The empty nanocarriers (EM) induced a very slight increase in GP in A-431 cells, with a weaker effect in HaCaT cells, implying that this nanocarrier selectively enhances the membrane order in cancer cells.

Treatment with podophyllotoxin (PPT) alone decreased the GP in A-431 cells, while HaCaT membranes were only slightly affected, highlighting the differential membrane-disruptive effect of the drug in cancerous versus non-cancerous cells. In contrast, when PPT was encapsulated in the nanocarrier (nPPT), both cell lines showed a significant increase in GP, with A-431 cells exhibiting a stronger response. This indicates the potentiated membrane-ordering effect of the drug-loaded formulation in cancer cells.

Etoposide (ETP), a chemotherapeutic agent, caused a marked reduction in GP exclusively in A-431 cells, suggesting that it fluidizes cancer cell membranes more effectively than those of non-cancerous cells. A similar but less pronounced effect on lipid disorder was observed for podophyllotoxin. In contrast, hydrogen peroxide (H₂O₂), an inducer of oxidative stress, increased the GP in both cell lines, with HaCaT cells showing a slightly more pronounced rise, which could have been due to the fact that non-treated A-431 cells intrinsically exhibited higher membrane lipid order than HaCaT ones. This points to a somewhat greater lipid packing response in the non-cancerous membrane environment.

In summary, these findings demonstrate that podophyllotoxin interacts with the membranes of HaCaT and A-431 cells in a cell type-specific manner. PPT decreased the GP in A-431 cells, while HaCaT membranes were only slightly affected, suggesting a differential membrane-disruptive effect in cancerous versus non-cancerous cells. In contrast, nPPT caused a significant increase in GP for both cell lines—greater in the cancerous cell line—indicating a membrane-ordering–based destabilizing effect.

The observed membrane-ordering effect induced by nPPT in A-431 cells has significant biological relevance with regard to cell survival and death. Enhanced lipid packing may disrupt essential cellular processes, including receptor mobility, endocytosis, and signal transduction pathways. In A-431 cells, which are characterized by the overexpression of the epidermal growth factor receptor (EGFR), membrane fluidity is crucial for proper receptor function and downstream signaling. Disruption of membrane dynamics can impair EGFR-mediated pathways, leading to reduced cell proliferation and increased susceptibility to apoptosis [25,26].

Although both etoposide and nPPT induced apoptosis in both cell lines, they exerted opposing effects on the membrane structural properties in the A-431 cell line, highlighting the complexity of the membrane dynamics in cytotoxic responses. Etoposide, a topoisomerase II inhibitor, induces DNA double-strand breaks and activates the intrinsic apoptotic pathway. Interestingly, its action is associated with decreased lipid order in A-431 cells. The observed overall decreased membrane lipid order can be also related to mitochondrial membrane permeabilization, allowing the release of pro-apoptotic factors such as cytochrome c and promoting the insertion and activation of Bcl-2 family proteins like Bax [27,28]. Thus, in this context, increased membrane fluidity is not merely a consequence of stress but may actively contribute to the execution of apoptosis by supporting mitochondrial outer membrane disruption. In contrast, podophyllotoxin, known to be a microtubule-disrupting agent, leads to mitotic arrest and apoptosis [5], yet it causes an increase in membrane order (i.e., decreased fluidity) when encapsulated. This membrane-ordering effect may interfere with the dynamics of survival signaling pathways, particularly those involving lipid rafts—specialized membrane microdomains that regulate receptor clustering and downstream signaling [29]. A more rigid membrane environment may promote the formation of death-inducing signaling platforms or prevent pro-survival receptor activation, thereby enhancing the sensitivity to apoptotic cues.

These findings underscore that the physical state of the membrane modulates, but does not solely determine, a cell’s fate. Both increased and decreased membrane lipid order can sensitize cells to apoptosis, depending on the cell type, its specific receptor profile, and the nature of the cytotoxic stimulus, and this may disrupt a range of essential cellular functions.

In summary, the treatment of A-431 cells with nPPT (PPT encapsulated in nanomicelles) increased GP, thus causing membrane ordering and interference with essential cellular processes in A-431 cells. This effect was detected solely in the cancerous A-431 cells in comparison with the normal HaCaT cells (Figure 10).

#### 2.2.6. FACS Analysis of Cell Cycle Arrest in Treated A-431 and HaCaT Cells

**Figure 11:**
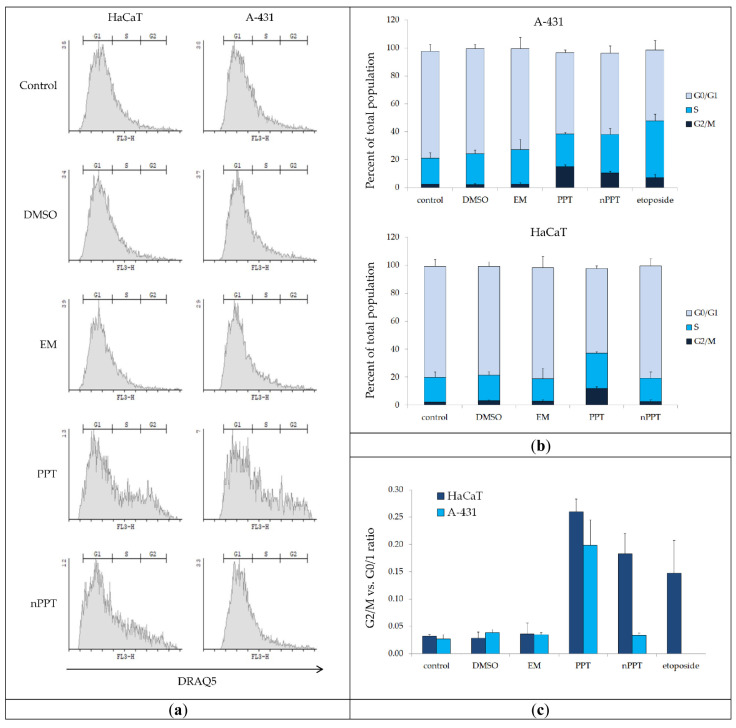
Cell cycle analysis by FACS. (**a**) Cells treated as indicated were stained with DRAQ5—a cell-permeant DNA-specific dye. The representative histograms show the cell cycle distribution based on the DNA content. (**b**) the stacked column graphs show the mean percentages of cells in the G0/G1, S, and G2/M phases of the cell cycle, measured as indicated in panel (**a**). Etoposide was used on HaCaT cells as a positive control for S-phase delay and G2/M-phase cell cycle arrest. (**c**) The graph shows the ratios of cells in the G2/M and the G0/G1 phases; error bars—standard deviation, *n* = 3.

The PPT treatments increased significantly the accumulation of A-431 and HaCaT cells in the S and G2/M phases (Figure 11). This was indicative of G2/M checkpoint activation and likely the induction of homology-directed DNA repair (HDR) mechanisms, which require the presence of sister chromatids [30]. In addition, the induction of cell cycle arrest at the S phase by PPT concurred with previously published data on PPT-treated human uveal melanoma MUM-2B cells [31]. For HaCaT cells, the PPT-induced G2/M vs. G0/G1 ratio was slightly higher as compared to A-431 cells (15.09% vs. 11.9%, respectively). More efficient G2 checkpoint/HDR activation may facilitate error-free DNA repair, leading to reduced cytotoxicity [32]. This might be related to the results regarding ROS generation and the caspase activity assays, confirming that, compared to HaCaT cells, the A-431 cells were more sensitive to the treatment.

Compared to PPT, the treatment with nPPT induced the slightly lower accumulation of HaCaT cells in the G2/M phase, and there was no significant G2/M checkpoint activation in A-431 cells (Figure 11). In accordance with the data from the GP test, PPT and nPPT had different effects on A-431. This cell line expresses abnormally high levels of epidermal growth factor receptor (EGFR), which plays a key role in cell proliferation, survival, and metastasis [33]. It is known from published data that the tyrosine kinase activity of EGFR increases with increasing cellular membrane fluidity [34]. As discussed above regarding the GP test, in the case of the nPPT treatment of A-431 cells, decreased membrane fluidity in A-431 cells was observed in comparison to the PPT treatment. The opposite effects of PPT and nPPT on the GP might be explained by the fact that nanoparticles with an average diameter of less than 50 nm and a zeta potential similar to that of the MPEG-*b-*PLA micelles used in this study can be internalized via caveolae-mediated endocytosis [35]. Caveolae are invaginated lipid rafts that are involved in numerous plasma membrane processes, including the regulation of EGFR signaling [36]. The interaction of the empty micelles with caveolae might be related to the slight increase in GP in A-431 cells. In addition, the significant increase in GP in A-431 cancer cells treated with PPT-loaded micelles (nPPT) might be associated with enhanced EGF receptor targeting, as it is known that caveolae are enriched with receptors of this family [37].

EGFR inhibitors have been shown to increase the G0/G1 vs. G2/M ratio [38,39]. In this regard, the targeted delivery of nanoencapsulated PPT to membrane domains enriched with EGFR may explain the reverted G0/G1 vs. G2/M ratio in nPPT-treated as compared to PPT-treated cells. Literature data show that PPT could inhibit EGFR and the proto-oncogene c-MET kinase in a concentration-dependent manner [11] and activate autophagic cell death [40]. Therefore, the modified release of PPT from the nanoparticles could influence the EGFR in A-431 in a different manner than does free PPT. The exact reasons for the different effects of PPT and nPPT on the regulation of the cell cycle in A-431 and HaCaT cells require more detailed investigations.

#### 2.2.7. Measurement of Genotoxic Effects of Tested Compounds on A-431 and HaCaT Cells by Comet Assay

**Figure 12:**
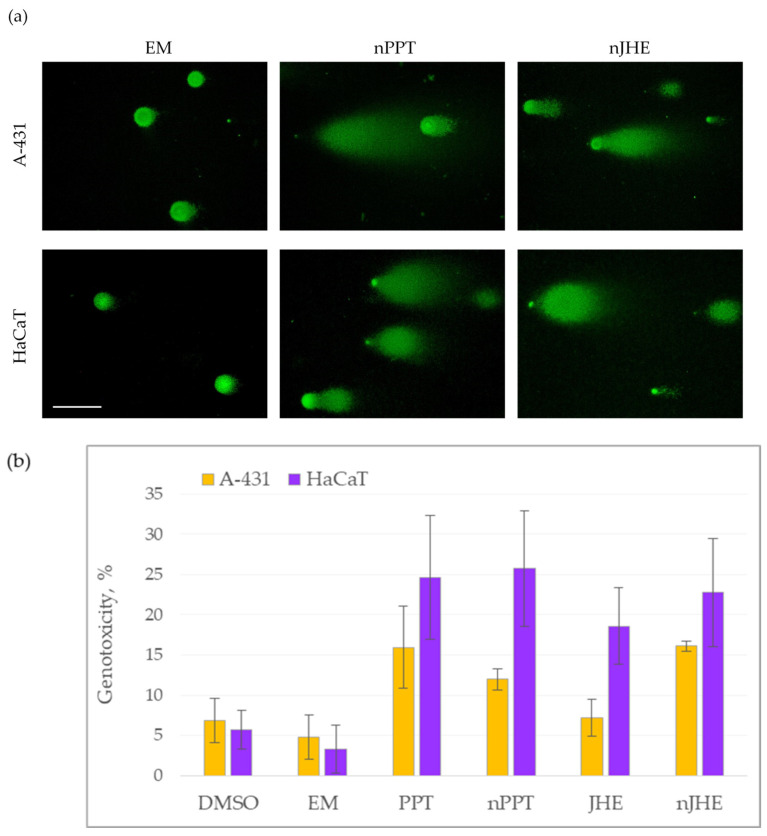
Genotoxicity of individual bioactive compounds (PPT and JHE) or with the loaded nanocarriers (nPPT and nJHE) in A-431 or HaCaT cells, as assessed by comet assay (single-cell gel electrophoresis). (**a**) Representative images of cells with undamaged DNA (intact nuclei) and cells with damaged DNA (comet objects). Scale bar 100 μm. (**b**). Assessment of the genotoxic effects of the tested compounds on A-431 and HaCaT cells treated for 24 h. For each sample, at least 100 objects were analyzed. Results are presented as mean ± SD of three biological replicates. Abbreviations: PPT—podophyllotoxin; nPPT—PPT-loaded micelles; JHE—*Juniperus horizontalis* leaf extract; nJHE—*Juniperus horizontalis* leaf extract-loaded micelles; DMSO—dimethyl sulfoxide, vehicle; EM—empty micelles.

The single-cell gel electrophoresis assay, also referred to as the comet assay, is a versatile and extremely sensitive technique for the detection and quantification of genomic DNA damage in eukaryotic cells [41,42,43]. To evaluate the genotoxic effects of EM, PPT, nPPT, JHE, and nJHE in A-431 and HaCaT cells, we performed the neutral variant of the comet assay. Representative images of intact nuclei (cells with undamaged DNA) and comet objects (cells with damaged DNA) are shown in Figure 12a. Empty micelles (EM) did not induce DNA damage in the two cell lines, while treatment with nPPT and nJHE resulted in the significant fragmentation of cellular DNA.

To quantify the genotoxicity of the studied bioactive ingredients without or with the loaded nanocarrier, at least 100 objects of each sample were analyzed. The results are presented in Figure 12b. The results showed that PPT and nJHE exhibited the highest and similar average genotoxicity in the A-431 cell line. As for HaCaT cells, all four compounds, PPT, nPPT, JHE, and nJHE, induced significant DNA damage and degradation compared to the EM and DMSO control. Out of the four compounds, PPT alone or nPPT caused the highest average genotoxicity in HaCaT cells. When comparing the two cell lines, it was obvious that, generally, HaCaT cells demonstrated higher sensitivity to the tested agents than A431 cells. The HaCaT cell line was more vulnerable to both the genotoxic (Figure 12) and cytotoxic activity (Table 2) of the studied bioactive compounds, consisting, respectively, of more significant DNA fragmentation and a lower median inhibitory concentration (IC_50_). Therefore, when considering the overall genotoxic effect, none of the examined substances at the tested concentrations seemed selective towards the tumor cell line.

Future preclinical studies are necessary to reveal whether in vivo application of the obtained water-dispersible PPT-containing polymer micelles can lead to their improved bioavailability and facilitated excretion, as well as reduced systemic toxicity in living organisms.

## 3. Materials and Methods

### 3.1. Chemicals

Podophyllotoxin (PPT, standard compound, ≥98%), MTT [3-(4,5-dimethylthiazol-2-yl)-2,5-diphenyltetrazolium bromide], *L*-glutamine, ethanol (96%), methanol (96%), acetone (ACS reagent, ≥99.5%), 2-propanol (ACS reagent, ≥99.8%), stannous octoate Sn(Oct)_2_ (92.5–100%), *D,L*-lactide, and methoxypolyoxyethylene were purchased from Sigma-Aldrich Fine Chemicals (St. Louis, MO, USA). Prior to use, *D*,*L*-lactide (LA) was recrystallized from toluene/ethyl acetate (95:5 *v/v*), and methoxypolyoxyethylene (MPEG-5K, Mn = 5000 g mol^−1^) was freeze-dried from toluene. Cell culture media, fetal bovine serum (FBS), and penicillin/streptomycin (Pen/Strep, 100×) were from Capricorn Scientific (Düsseldorf, Germany). Hoechst 33342 stain (B2261) was delivered from Merck & Co., Inc. (Rahway, NJ 07065, USA).

### 3.2. Plant Material and Preparation of Extracts

*Juniperus horizontalis* (creeping juniper, specimen number 1164-56-A) and *Juniperus virginiana* ‘Grey Owl’ (Grey Owl juniper, specimen number 1136-61-A) wings were collected in February 2024 from the Arnold Arboretum, Harvard University, Boston, MA, USA. The plant material was dried for a week at room temperature until a constant weight was reached and kept in the freezer in closed vacuum bags until analyses. The extracts were obtained by a previous procedure [4]. Briefly, dried ground leaves (5 g) of the corresponding juniper species were suspended in methanol (50 mL, 80% *v/v*) in a closed Erlenmeyer flask. The extraction was carried out for 1.5 h in a shaker water bath at room temperature, and the extract was collected by filtration. This procedure was repeated two additional times. After this, the solvents from the combined extracts were evaporated in vacuo, the chlorophyll-containing dark green oil was removed, and the resulting water-containing extract was lyophilized and stored in the freezer until analyses.

### 3.3. MPEG-b-PLA Block Copolymer Synthesis

The MPEG-*b*-PLA block copolymer was synthesized according to a previously described procedure, with modifications [20]. MPEG-5K (2 g, 0.4 mmol) was freeze-dried from a toluene solution. Then, LA (1.95 g, 13.6 mmol) was added and the mixture was dried under a high vacuum for 1 h, followed by flushing the flask with argon. Finally, 13 µL Sn(Oct)_2_ (0.04 mmol) was added by syringe. The polymerization proceeded in bulk at 125 °C (oil bath) for 2 h. The crude product was extracted with 2-propanol, filtered, and dried to yield an MPEG-*b*-PLA diblock copolymer. The molar mass of the MPEG block was 5000 g mol^−1^ (DP_n_ = 114), and the molar mass of the PLA block was 4300 g mol^−1^ (DP_n_ = 30). ^1^H NMR (400 MHz, CDCl_3_): δ (ppm) 5.16 [C*H*-(CH_3_)-O], 4.29 [C*H*-(CH_3_)-OH], 3.65 (O-C*H*_2_-C*H*_2_-O), 3.38 (C*H*_3_-O), 1.56 [CH-(C*H*_3_)-O], 1.46 [CH-(C*H*_3_)-OH]. Yield: 3.5 g, 90%.

### 3.4. Preparation of Micelles

The solvent evaporation method was used for the preparation of the micelles. Briefly, the MPEG-b-PLA block copolymer was dissolved at room temperature in acetone at a concentration of 10 mg mL^−1^. Then, the polymer solution (1 mL) was added dropwise to approximately 8 mL of ultrapure water under stirring (1000 rpm). After the evaporation of the acetone (vacuum evaporator), the concentrations of the micellar dispersions were adjusted to 1 mg mL^−1^ by the addition of ultrapure water. Prior to analysis, the polymer dispersions were filtered through a Millipore^®^ 0.45 μm disk filter (Merck Millipore, Burlington, MA, USA).

### 3.5. Drug Loading of Micelles

For the preparation of loaded micelles, podophyllotoxin (PPT) or juniper extracts (JHE and GO) were firstly dissolved in ethanol at a concentration of 1 mg mL^−1^ and then 1 mL of the solution was added slowly to 10 mL (1 mg mL^−1^) of the stirred MPEG-b-PLA micelle dispersion. After ethanol removal (rotary evaporator), the concentration was adjusted to 1 mg mL^−1^ (micelles to PPT or juniper extracts—10:1 *w*/*w*). The micellar dispersions were filtered (0.45 µm), lyophilized, and then resuspended in ethanol for UV/Vis spectroscopic analysis at a wavelength of 290 or 277 nm for PPT or juniper extracts, respectively. The extinction coefficients, namely ε = 4432 M^−1^ cm^−1^ (λ_max_ = 290 nm) for PPT, ε = 4.9925 L g^−1^ (λ_max_ = 277 nm) for juniper extract JHE, and ε = 5.0876 L g^−1^ (λmax = 277 nm) for juniper extract GO, were obtained from the calibration curve in ethanol. The encapsulation efficiency (EE) and loading capacity (LC) were calculated according to the following equations:

EE (wt%) = (S_m_ /S_0_) × 100, (1)

where S_m_ is the amount of active substance in micelles, and S_0_ is the total initial amount of active substance;

LC (wt%) = (S_m_/M) × 100, (2)

where S_m_ is the amount of active substance in micelles, and M is the amount of micelles.

### 3.6. In Vitro Drug Release Profiles

A total of 10 mL of the PPT- or juniper extract-loaded copolymer micelles (1 mg mL^−1^) was transferred to cellulose dialysis membrane tubing (MWCO 12,000–14,000 Da) that had been pretreated with distilled water for 12 h prior to use. The loaded bag was immersed in 100 mL of release media comprising phosphate buffer (pH 7.4) and ethanol 9:1 (*v*/*v*). The system was kept at 37 °C and stirred at a rate of 100 rpm. At predetermined time intervals, 1 mL samples were withdrawn from the release media, and the amount of the corresponding active substance was detected by UV/Vis spectroscopy (λ_max_ = 287 nm for PPT and λ_max_ = 275 nm for both JHE and GO extracts). After each sample’s withdrawal, an equal volume of fresh medium was added in order to maintain the sink conditions. The results are presented as the percentage of released drug vs. time. Each experiment was run in triplicate.

### 3.7. Characterization Methods

^1^H-NMR spectra were recorded on a Bruker Avance NEO spectrometer (Billerica, MA, USA) at 400 MHz. Size exclusion chromatography (SEC) was run on a HPLC Shimadzu Nexera XR (Kyoto, Japan) instrument in tetrahydrofuran at a flow rate of 1 mL min^−1^. The polymers’ molar mass characteristics were determined using an RID-20A differential refractive index detector on the following set of columns: 10 μm PL gel mixed-B, 5 μm PL gel 500 Å, and 50 Å. Narrow-dispersity polystyrene (PS) standards were applied for the instrument’s calibration and for the calculations. The morphology data were acquired on a HRTEM JEOL JEM-2100 (200 kV) transmission electron microscope (TEM) (JEOL, Peabody, MA, USA) equipped with a CCD camera GATAN Orius 832 SC1000 (Pleasanton, CA, USA) and the GATAN Microscopy Suite 3.4 software. UV/Vis spectra were recorded on a DU 800 Beckman Coulter spectrometer (Beckman Coulter, Inc., Brea, CA, USA). The Z-average diameter, polydispersity index (PdIs), and zeta potential values of empty and active substance-loaded micelles were determined by DLS using the NanoBrook Plus PALS instrument (Brookhaven Instruments, New York, NY, USA), equipped with a 35 mW solid-state laser operating at λ = 660 nm and at a scattering angle of 90°.

The particles’ hydrodynamic diameters (d_H_) were determined according to the Stokes–Einstein equation:

d_H_ = kT/(3πηD), (3)

where k is Boltzmann’s constant, T is the absolute temperature, η is the viscosity, and D is the diffusion coefficient.

The ζ-potentials were calculated from the obtained electrophoretic mobility via the Smoluchowski equation:

ζ = 4πημ/ε,(4)

where η is the solvent viscosity, μ is the electrophoretic mobility, and ε is the dielectric constant of the solvent.

All measurements were carried out in triplicate at 25 °C; size and zeta potential measurements were recorded as averages of 3 and 20 runs, respectively.

### 3.8. Cell Culture Conditions

Two adherent human skin cell lines were chosen for the present study—the tumorigenic cell line A-431 (cutaneous non-melanoma epidermoid squamous cell carcinoma), expressing high levels of EGFR, and the non-tumorigenic cell line HaCaT (spontaneously transformed and immortalized keratinocytes retaining the capacity to undergo differentiation). They were purchased from the CLS Cell Lines Service (GmbH, Eppelheim, Germany). The cells were maintained according to the recommendations of the biobank and as previously described in the literature [44]. In brief, the cells were cultured in the cell culture medium DMEM-HG with 4.5 g/L glucose, supplemented with 10% heat-inactivated fetal bovine serum (FBS), *L-*glutamine (2 mM), and Pen/Strep (penicillin G sodium 10^5^ units/L and streptomycin sulfate 100 mg/L, final concentrations) in a humidified incubator (Panasonic MCO-18AC, Panasonic Healthcare Ltd., Oizumi-Machi, Japan) at 37 °C and 5% CO_2_. As adherent cell lines, they were trypsinized 1–2 times per week (dilution 1:8), which allowed the cells to remain in the log phase.

### 3.9. MTT Assay for Antiproliferative Activity Determination

The assay for the detection of the antiproliferative activity of the studied bioactive components and the corresponding loaded nanocarriers was performed as described previously [45]. A-431 cells were seeded at a density of 1.05 × 10^5^ cells/mL, and HaCaT cells were seeded at a concentration of 1.50 × 10^5^ cells/mL. Three exposure times were tested—24, 48, and 72 h—and the inhibitory concentration 50% (IC_50_), also known as the mean or half-maximal inhibitory concentration, was calculated from the dose–response curves for each sample. This seeding concentration was also used for the other assays.

### 3.10. Hoechst Assay for DNA Staining During Apoptosis Induction in Cancer and Normal Cells

Hoechst dye was used to visualize DNA during the middle stage of apoptosis induction by PPT-containing bioactive components or by the corresponding loaded nanocarriers. The cell lines A-431 and HaCaT were seeded in the same way as described for the MTT assay, except that a 50 µL suspension of the corresponding cell line was seeded per well using black 96-well plates with a clear bottom. The cells were treated with the bioactive components at concentrations within the ranges of their IC_50_ values, as follows: JHE and nJHE were used at concentrations of 0.4 and 0.2 µg/mL of the active ingredient; PPT and nPPT at 0.006 and 0.003 µg/mL of the active ingredient; and EM at 1 and 5 µg/mL. After different periods of exposure (24, 30, and 48 h; see Supplementary Table S1), Hoechst 33342 (cat. № B2261, Merck & Co., Inc., Rahway, NJ 07065, USA) (5 µg/mL) was added, and, after incubation (20 min) in the dark, the cells were observed under an inverted fluorescence microscope (BOE 5000.930, mode BIB100, BOECO, Hamburg, Germany). The results were photodocumented microscopically (B-CAM16, BOE 1900.16000, BOECO, Hamburg, Germany).

### 3.11. Caspase-3 and Caspase-7 Assays

A 96-well plate format was chosen, and it was seeded as described for the MTT assay. The cells were treated with PPT and nPPT, with the PPT content equal to the IC_50_ at 48 h exposure of the PPT in the loaded nanocarrier (nPPT) on the tumor cell line A-431 (0.005 µg/mL PPT content). The vehicle dimethyl sulfoxide (DMSO), in which the free components were dissolved, and EM were also used, in the same concentrations as in the solubilized or loaded samples. In addition, etoposide, a compound known to induce apoptosis, was used as a positive control and for comparison (with a concentration of 50 µM). Treatment was carried out in duplicate. After 24 hours of exposure, the caspase-3 and caspase-7 activity was measured using the Caspase-Glo^®^ 3/7 Assay (# G8090 Promega Corporation, Madison, WI, USA), according to the manufacturer’s protocol. The Caspase-Glo^®^ 3/7 Buffer was mixed with the Caspase-Glo^®^ 3/7 substrate (both equilibrated to room temperature) to form the reagent. The plate was equilibrated to room temperature. The reagent (100 μl) was added to each well of the plate, taking measures to avoid contact between the pipette tips and the wells containing different samples so as to avoid cross-contamination. The contents of the wells were mixed using a plate shaker at 300–500 rpm for 30 s. The plate was incubated at room temperature for 45 min, and the lysed contents of each well were transferred to a white-walled 96-well plate. The luminescence of each sample was detected in a plate-reading luminometer. For blank samples, pure culture medium, culture medium with DMSO, and culture medium with micelles (both agents with the same concentrations as in the treated samples) were used. Due to the luminescence of the fetal calf serum in the medium, the cell-free blank values were subtracted from the values of the corresponding samples.

### 3.12. Intracellular ROS Generation Assay

The intracellular ROS of the samples were measured using the Fluorometric Intracellular ROS Kit (# MAK143, Sigma-Aldrich, St. Louis, MO, USA). Cells were seeded in black 96-well plates with a clear bottom (50 µL cell suspension per well). After 24 h incubation, 55 µL of the Master Reaction Mix was added to each well. The mix consisted of assay buffer and ROS detection reagent stock solution reconstituted in DMSO (1:500). Then, the plate was incubated for one hour under standard conditions. Next, the plate was treated with PPT or nPPT, at a 0.005 µg/mL concentration of the active ingredient, with etoposide at a concentration of 50 µM, with the vehicles EM and DMSO, and with 0.35% H_2_O_2_ used as a positive control. For blank samples, the corresponding amount of buffer was used. The plate was incubated for 30 h under standard conditions. The fluorescence was measured at 490_ex_/520_em_ nm.

### 3.13. Membrane Lipid Order Assessment

The Laurdan staining and spectroscopy were performed using Laurdan (6-dodecanoyl-2-dimethylaminonaphthalene; Thermo Fisher) in order to assess the membrane lipid order. A 0.7 mM stock solution of Laurdan in DMSO was used to achieve a final concentration of 3.5 µM Laurdan in the cell suspensions. Cells were seeded in black, clear-bottom 96-well plates (suitable for fluorescence measurements) at a density of 1 × 10^4^ cells per well and allowed to adhere for 24 h prior to treatment. Cells (control) were treated with the vehicle DMSO, the empty nanocarrier (EM), podophyllotoxin (PPT), nanoencapsulated PPT (nPPT), etoposide (ETP), or hydrogen peroxide (H_2_O_2_), similarly to the ROS generation assay described above. Then, the cells were washed gently with phosphate-buffered saline (PBS) and incubated with Laurdan-containing medium for 60 min at 37 °C in the dark. After incubation, cells were washed twice with PBS to remove unincorporated dye.

The fluorescence intensity was measured using a TECAN Infinite M200 Pro microplate reader. Laurdan was excited at 355 nm, and emission was collected at two wavelengths: 440 nm (I_440_, representing emission in ordered membrane environments) and 490 nm (I_490_, reflecting emission in disordered, more polar environments). The Laurdan generalized polarization (GP) was calculated using the following equation: GP = (I440 − I490)/(I440 + I490), where I440 and I490 represent the fluorescence intensities at 440 nm and 490 nm, respectively. The GP is given as average values from at least four independent replicates per condition, as each replicate was measured twenty times. Data were analyzed using Origin (v9.0).

### 3.14. Fluorescence-Activated Cell Sorting (FACS) for Cell Cycle Analysis

Cells were seeded in 24-well plates and treated with PPT and nPPT, with a concentration of the active ingredient (PPT) equal to the IC_50_ at 48 h exposure of the active ingredient in the loaded nanocarrier (nPPT) on the tumor cell line A-431 (0.005 µg/mL PPT). After 40 h of exposure, cells were detached with a 100 µL solution of trypsin (for HaCaT) or Accutase^®^ (#ACC-1B, Capricorn Scientific, Ebsdorfergrund, Germany) (for A-431). The detachment agent was neutralized by the addition of 150 µL culture medium. Then, 10 μM of DRAQ5 (# 65-0880-96, Invitrogen, Waltham, MA, USA) was added to each sample and they were incubated at 37 °C for 30 min. Samples were diluted appropriately for the instrument (with 150 µL PBS) and were analyzed with a BD FACSCalibur™ Flow Cytometer, and the data were processed with the Cyflogic 1.2.1 software (https://www.cyflogic.com accessed on 1 March 2025).

### 3.15. Comet Assay for DNA Fragmentation Detection

The cell lines A-431 and HaCaT were seeded in 6-well plates, and, after 24 h, they were treated with a concentration of the active ingredient of the samples (JHE or PPT) equal to the IC_50_ of 48 h exposure of the active ingredient in the loaded nanocarriers (nJHE or nPPT) on the tumor cell line A-431. The tested concentrations were 0.4 μg/mL for nJHE and 0.005 μg/mL for PPT. The vehicles EM (5 μg/mL) and DMSO were also used, in the same concentrations in which they were administrated in the solubilized or loaded samples.

To measure the genotoxic effects of the studied compounds, the single-cell gel electrophoresis assay (comet assay) in neutral conditions was performed. The comet assay was carried out generally as it was described in the literature [43]. Following 24 h exposure to the agents, adhered cells were collected from the cultivating plate and centrifuged at 5000 rpm (2380× *g*) for 5 min. After washing the cells with 1 mL of phosphate-buffered saline (1X PBS, pH 7), they were resuspended in 500 μL of (1X PBS, pH 7. An appropriate aliquot of the cell suspension was mixed with low-melting agarose (Thermo Scientific^TM^, Thermo Fisher Scientific, Waltham, MA, USA) to a final concentration of agarose 0.7% (*w*/*w*) and spread on a microscopic slide. For the polymerization of the agarose, the slides with gel-embedded cells were kept at 4 °C for 5 min and then incubated in lysis buffer (146 mM NaCl, 10 mM Tris pH 8.0, 30 mM EDTA pH 8.0, 0.1% N-lauroylsarcosine) at 4 °C for 20 min. Next, microgel-containing slides were incubated in TBE buffer (44.5 mM Tris, 44.5 mM H_3_BO_3_, 1 mM EDTA) twice for 15 min each at 4 °C. Electrophoresis was performed at 0.45 V/cm in TBE buffer, at 4 °C for 10 min. For the dehydration of microgels, the slides were submerged alternately in 75% and 95% ethanol for 5 min each. Finally, the gels on the slides were left to air-dry. The cellular DNA content was stained with the fluorescent dye SYBR Green I (Roche Diagnostics GmbH, Mannheim, Germany) at 3000X dilution. The SYBR Green I fluorescently stained objects (nuclei, comets, apoptotic bodies) were visualized using a Leitz epifluorescent microscope, Leitz Orthoplan, using a 450–490 band-pass filter. Images were documented by a Levenhuk M1400 Plus (Levenhuk Inc., Tampa, FL, USA) camera, using the software provided by the camera manufacturer. For the evaluation of DNA damage, the number of objects without DNA fragmentation (intact nuclei) and the number of objects with the degradation of DNA (comets and apoptotic bodies) were determined. Genotoxicity was represented as the percentage of objects with DNA damage.

### 3.16. Data Processing and Statistics

A non-linear regression analysis (curve fit, GraphPad Prism 9 software, GraphPad Software Inc., San Diego, CA, USA) was used to calculate the IC_50_ values from the MTT assays using sigmoidal concentration–response curves. For statistical analysis, Student’s *t*-test was used, with *p* ≤ 0.05 set as the lowest level of statistical significance for the MTT, caspase 3/7, ROS, GP, and FACS experiments. The 95% confidence interval (CI) of the IC_50_ values was derived from the dose–response curves, with a regression coefficient (R^2^) in the range of 0.88–0.99. One well per sample and two for the control were used for the comet assay. Comet assay measurements are presented as a result of three experiments. Treatments for the other experiments were carried out in quadruplicate (MTT, ROS, and GP assays) and in duplicate for the rest of the assays. Statistical comparisons between treatment groups were performed using one-way ANOVA for the caspase-3/-7, ROS, and GP assays, followed by Tukey’s post hoc test, and the results were expressed as the mean ± standard deviation (SD). One-way and two-way ANOVAs were applied for the FACS experiments, followed by Tukey’s and Sidak’s post hoc tests, respectively (Supplementary Tables S2–S6).

## 4. Conclusions

Nanotechnology offers alternative approaches to the discovery of efficient and less toxic therapeutic agents. The loading of toxic hydrophobic bioactive agents in the cores of amphiphilic nanocarriers leads to water-dispersible products with expected increased bioavailability in the blood, facilitated excretion from treated organisms, and therefore reduced toxicity. The present study was focused on the construction and physicochemical characterization of new polymer nanosized micelles loaded with the anticancer drug precursor podophyllotoxin (PPT) or PPT-containing juniper leaf extracts and the study of their antineoplastic activity.

A well-defined, biodegradable, and biocompatible amphiphilic MPEG-*b*-PLA diblock copolymer was synthesized and evaluated as a nanocarrier for podophyllotoxin and two podophyllotoxin-containing *Juniperus horizontalis* (JHE) and Grey Owl (GO) juniper leaf extracts. The copolymer self-assembled in aqueous media into nanosized micelles with an average diameter of around 45 nm. The preformed micelles were successfully loaded with podophyllotoxin or the two PPT-containing juniper extracts, which were stable for at least one month. After loading with the active substances, the average diameters of the MPEG-*b-*PLA nanoparticles slightly increased to 46.00, 51.63, and 48.68 nm for PPT-, JHE-, and GO-loaded micelles, respectively. The nanocarriers’ physicochemical properties, drug-loading efficiency, drug-loading capacity, physical stability, and in vitro release drug profiles were determined. The highest encapsulation efficiency (EE) was obtained for PPT (98 wt%), followed by GO (83 wt%) and JHE (74 wt%).

Antineoplastic activity was evaluated in A-431 epidermoid carcinoma cells and HaCaT normal keratinocytes. As *Juniperus horizontalis* extract (JHE) showed higher antiproliferative activity than Grey Owl extract, JHE was chosen for in-depth bioactivity analyses. The results of the MMT assay showed that the loaded polymer micelles (nPPT, nJHE) retained the high antiproliferative activity of the starting hydrophobic bioactive components, PPT and JHE. The most effective antiproliferative activity was detected after 48 h of the treatment of A-431 and HaCaT cells with the PPT component-loaded micelles. The studied juniper extract-loaded micelles were less cytotoxic than the PPT-loaded nanoparticles. The antiproliferative activity of nJHE (IC_50_ 0.4 μg/mL after 48 h treatment of A-431 cells) was sufficient to consider it as an effective potential anticancer agent for the treatment of epidermoid carcinoma.

The Hoechst assay visualized DNA during the middle stage of apoptosis and revealed chromatin and nuclear condensation and shrinkage, as a hallmark of apoptosis, induced in the treated A-431 and HaCaT cells by the PPT- and JHE-loaded MPEG-*b*-PLA micelles.

As a result of the various cellular processes induced by the PPT component-loaded nanoparticles, effector caspase-3 and caspase-7 activation, as well as reactive oxygen species (ROS) induction, showed good selectivity, being more efficient in the A-431 epidermoid carcinoma cells than in the normal HaCaT keratinocytes.

Considering the genotoxic effects, as analyzed by the comet assay, of the treated cell lines, the examined PPT-containing polymer micelles induced significant genotoxicity in the tumor A-431 cells, whereas normal HaCaT cells were more sensitive to this effect.

Regarding membrane fluidity, PPT decreased the GP in A-431 cells, while HaCaT membranes were only slightly affected, suggesting a differential membrane-disruptive effect of the drug in cancerous versus non-cancerous cells. In contrast, nPPT caused a significant increase in GP for both cell lines, being even greater in the A-431 cells, suggesting a membrane-ordering effect. Both increased and decreased membrane lipid order may interfere with essential cellular processes; thus, it can induce apoptosis, depending on the cellular type and its specific receptors.

In agreement with previous studies, in the current trial, the PPT treatment induced cell cycle arrest in the S and G2/M phases for both HaCaT and A-431 cells. In contrast, the two cell lines reacted differently to treatment with PPT-loaded micelles in regard to cell cycle regulation. Again, as in the GP test, PPT and nPPT had different effects on A-431 cells. This cell line was characterized by the overproduction of epidermal growth factor receptor (EGFR) [33], and the cells were unusual in their response to physiological concentrations of EGF [46]. As previously reported, EGFR is a target of PPT derivatives [40,47,48], and it is well known that growth factors and their receptors play essential roles in cell cycle regulation [49].

Therefore, it will be particularly interesting in the future to investigate the potential involvement of EGFR in the response of A-431 cells to micelle-loaded PPT in more rigorous trials.

In summary, a biodegradable and biocompatible amphiphilic MPEG-*b-*PLA diblock copolymer was characterized as a nanocarrier for podophyllotoxin and podophyllotoxin- containing juniper leaf extracts. MTT tests revealed the effective antiproliferative properties of the obtained PPT component-loaded nanomicelles in the studied cell lines. The comet assay and Hoechst staining revealed DNA fragmentation induction by the PPT-loaded nanocarriers in A-431 epidermoid carcinoma cells and HaCaT keratinocytes. The Laurdan generalized polarization method revealed that the treatment of A-431 cells with nPPT (PPT encapsulated in nanomicelles) increased the GP and thus caused membrane ordering and interference with essential cellular processes selectively in the cancerous A-431 cells in comparison with the normal HaCaT cells. In addition, selective apoptosis induction by effector caspase-3/-7, ROS induction, and other effects were determined and analyzed in the studied epidermoid carcinoma cells.

In conclusion, PPT-containing MPEG-*b*-PLA-micelles retained the antiproliferative activity and apoptosis-inducing potential of the starting hydrophobic bioactive components in the studied tumor and normal cell lines. Future studies should be performed to study the long-term stability of the PPT component-loaded nanomicelles under various storage conditions, their in vivo toxicity, and the pharmacokinetics/pharmacodynamics properties of the PPT-containing polymer micelles due to their improved dispersibility in water, their expected improved bioavailability, and their facilitated excretion from the treated living organisms, in comparison with the starting individual hydrophobic bioactive components.

The newly obtained PPT-containing nanoparticles have potential applications for the treatment of malignant diseases in the prospective nanomedicine.

## Figures and Tables

**Table 1:** Characteristics of empty and bioactive component-loaded block copolymer micelles.

Code	d ^a^ (nm)	PdI^a^	ζ ^a^ (mV)	EE ^b^ (%)	LC ^b^ (%)
**EM**	45.45± 0.61	0.169	1.21± 3.40	-	-
**nPPT**	46.00 ± 1.06	0.145	3.14 ± 3.79	98	8.9
**nJHE**	51.69 ± 0.93	0.205	0.37 ± 1.91	74	7.4
**nGO**	48.68 ± 0.16	0.166	0.17 ± 0.65	83	8.3

^a^ Average micelle diameters (d), size distributions (PdI), and zeta potentials (ζ) were obtained by dynamic light scattering measurements. ^b^ Encapsulation efficiency (EE) and loading capacity (LC) were determined spectroscopically. Abbreviations: EM-empty micelles; nPPT–PPT loaded block copolymer micelles; nJHE–*J. horizontalis* leaf extract loaded micelles; nGO–Grey Owl juniper leaf extract loaded micelles.

**Table 2:** Antiproliferative activity of podophyllotoxin (nPPT) and *Juniperus horizontalis* extract (nJHE) loaded in MPEG-*b*-PLA-based nanocarriers, in comparison with the corresponding individual bioactive components (PPT and JHE), in A-431 epidermoid carcinoma cells and HaCaT normal keratinocytes after 24, 48, and 72 h exposure.

Cell Lines and Parameters	Treatment Time of the Cell Lines with the Corresponding Bioactive Components					
**A-431** Parameter	**PPT**			**nPPT**		
	**24 h**	**48 h**	**72 h**	**24 h**	**48 h**	**72 h**
Hillslope	−0.9109	−3.904	−5.357	−0.8062	−1.810	−3.941
R^2^	0.9173	0.9641	0.9872	0.9225	0.9668	0.9844
IC_50_ [µg/mL]	0.003904	**0.003250**	0.003272	0.007020	**0.005323**	0.007071
CI 95%	0.003131–0.004868	0.002971–0.003555	0.003069–0.003490	0.005682–0.008673	0.004648–0.006095	0.006663–0.007504
**A-431** Parameter	**JHE**			**nJHE**		
	**24 h**	**48 h**	**72 h**	**24 h**	**48 h**	**72 h**
Hillslope	−4.147	−5.815	−6.145	−2.090	−1.948	−4.518
R^2^	0.9393	0.9659	0.9774	0.9386	0.9298	0.9665
IC_50_ [µg/mL]	0.7649	**0.6909**	0.7419	0.5012	**0.3895**	0.4612
CI 95%	0.6904–0.8442	0.6218–0.7617	0.6822–0.8211	0.4375–0.5712	0.3282–0.4550	0.4291–0.4971
**HaCaT** Parameter	**PPT**			**nPPT**		
	**24 h**	**48 h**	**72 h**	**24 h**	**48 h**	**72 h**
Hillslope	−0.6353	−1.138	−2.232	−0.6441	−1.322	−1.156
R^2^	0.9641	0.9728	0.9418	0.9348	0.9573	0.9364
IC_50_ [µg/mL]	0.006400	**0.002831**	0.005868	0.009696	**0.004048**	0.004963
CI 95%	0.005588–0.007330	0.002520–0.003181	0.004896–0.007032	0.007945–0.01183	0.003530–0.004641	0.004167–0.005910
**HaCaT** Parameter	**JHE**			**nJHE**		
	**24 h**	**48 h**	**72 h**	**24 h**	**48 h**	**72 h**
Hillslope	−0.7868	−1.407	−2.101	−0.7532	−1.374	−1.264
R^2^	0.9090	0.9790	0.9587	0.9395	0.9537	0.9398
IC_50_ [µg/mL]	0.9809	**0.2724**	0.3145	0.4451	**0.1597**	0.1686
CI 95%	0.7972–1.228	0.2479–0.2992	0.2793–0.3524	0.3745–0.5318	0.1361–0.1858	0.1407–0.2002

Abbreviations: IC_50_—half-maximum growth-inhibitory concentration derived from the MTT assay dose–response curve of the corresponding bioactive component after the treatment of A-431 and HaCaT cells; CI—95% confidence interval of the IC_50_ value; R^2^—regression coefficient.

**Table 3:** Cytotoxicity of MPEG-*b*-PLA-based empty micelles (EM) after treatment of A-431 epidermoid carcinoma or HaCaT normal keratinocyte cells.

EM [μg/mL]	A-431 Epidermoid Carcinoma % Viable Cells			HaCaT Normal Keratinocytes % Viable Cells		
	24 h	48 h	72 h	24 h	48 h	72 h
1	89	84	93	100	96	85
5	85	83	92	87	84	78
10	86	82	88	85	77	67
20	72	75	83	79	72	62
40	71	67	66	64	64	51

**Table 4:** Hoechst assay for DNA staining of A-431 and HaCaT cells treated for 48 h with PPT-containing bioactive components.

Sample	HaCaT	A-431
Untreated control cells	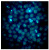	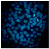
DMSO	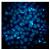	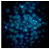
EM, 5 μg/mL	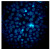	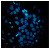
JHE, 0.4 μg/mL	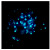	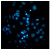
nJHE, 0.4 μg/mL	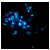	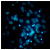
PPT, 0.006 μg/mL	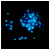	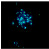
nPPT, 0.006 μg/mL	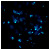	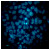

Abbreviations: PPT—podophyllotoxin; nPPT—PPT-loaded nanosized micelles; JHE—*Juniperus horizontalis* leaf extract; nJHE—*J. horizontalis* leaf extract-loaded micelles; DMSO—dimethyl sulfoxide, vehicle; EM—empty micelles; untreated cells—control.

## Data Availability

Data were included in the manuscript.

## Supplementary Materials

The following supporting information can be downloaded at: https://www.mdpi.com/article/10.3390/ijms26115167/s1.
