# Supplementary material for: Antineoplastic Activity of Podophyllotoxin and Juniper Extracts Encapsulated in MPEG-b-PLA Diblock Copolymer Micelles in Cutaneous Squamous Carcinoma Cells
Source: Int J Mol Sci. 2025 May 28;26(11):5167. doi: 10.3390/ijms26115167 (PMC12154351; doi:10.3390/ijms26115167)
Supplement: Supplementary file 1 [file ijms-26-05167-s001.zip › Suppl Table S1 Hoechst assay.pdf]

Supplementary Table S1. Hoechst assay of A-431 epidermoid carcinoma cells and HaCaT keratinocytes treated with podophyllotoxin (nPPT) - or *Juniperus horizontalis* leaf extract (nJHE) loaded MPEG-*b*-PLA diblock copolymer micelles.

A. Treatment for 24 h

Sample	HaCaT	A-431
Untreated control cells	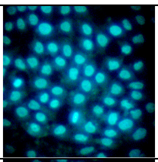	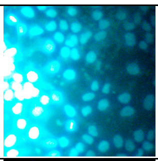
Empty micelles, 5 µg/mL	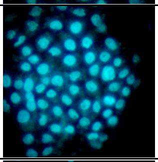	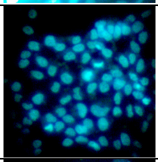
JHE, 0.4 µg/mL	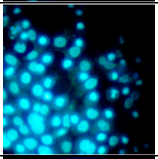	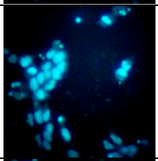
nJHE, 0.4 µg/mL	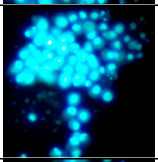	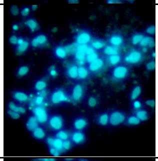
PPT, 0.003 µg/mL	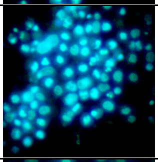	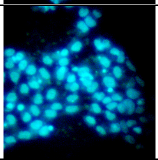
nPPT, 0.003 µg/mL	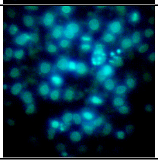	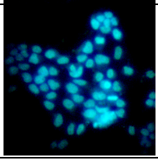

B. Treatment for 30 h

Sample	HaCaT	A-431
Untreated control cells	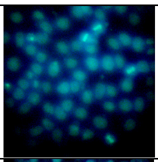	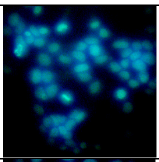
Dimethyl sulfoxide (DMSO), vehicle	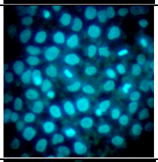	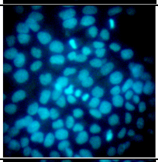
Empty micelles, 5 µg/mL	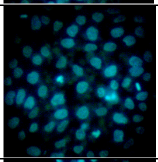	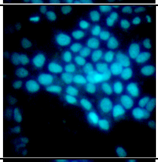
JHE, 0.4 µg/mL	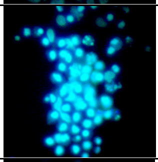	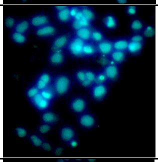
JHE, 0.2 µg/mL	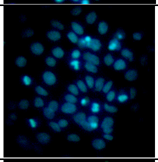	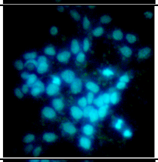
nJHE, 0.4 µg/mL	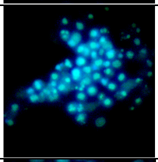	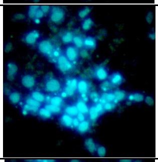
nJHE, 0.2 µg/mL	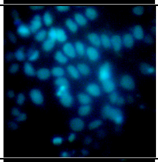	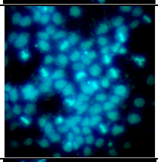
PPT, 0.006 µg/mL	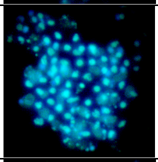	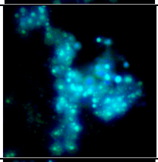
PPT, 0.003 µg/mL	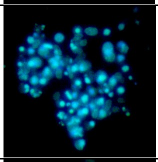	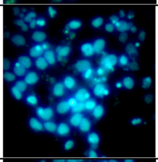
nPPT, 0.006 µg/mL	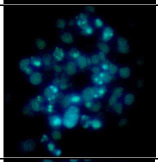	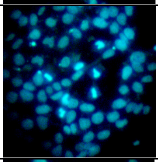
nPPT, 0.003 µg/mL	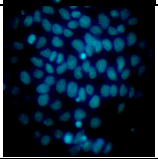	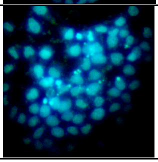

C. Treatment for 48 h

Sample, µg/mL	HaCaT	A-431
Untreated control cells	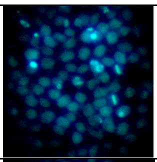	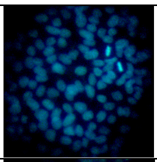
DMSO	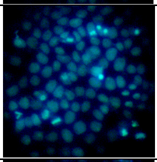	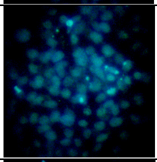
Empty micelles,<br>5 µg/mL	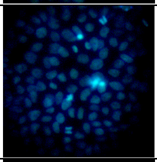	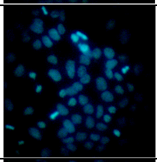
JHE,<br>0.4 µg/mL	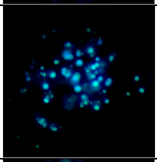	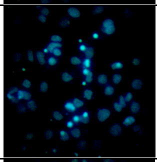
JHE,<br>0.2 µg/mL	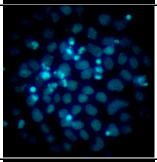	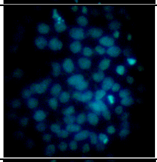
nJHE,<br>0.4 µg/mL	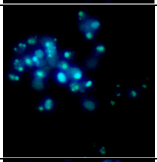	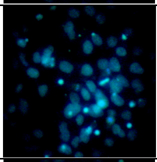
nJHE,<br>0.2 µg/mL	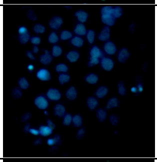	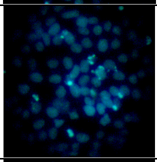
PPT,<br>0.006 µg/mL	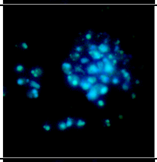	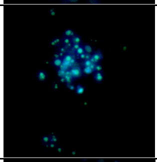
PPT,<br>0.003 µg/mL	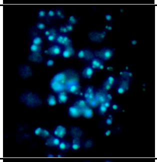	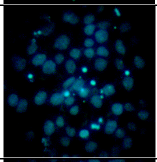
nPPT,<br>0.006 µg/mL	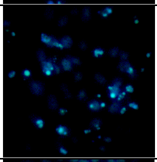	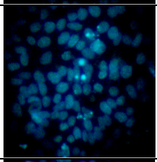
nPPT,<br>0.003 µg/mL	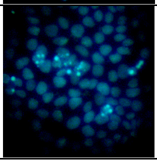	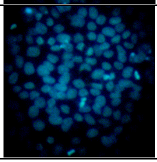

Abbreviations: PPT (podophyllotoxin), nPPT (PPT-loaded nanosized micelles), JHE (*Juniperus horizontalis* leaf extract), nJHE (*J. horizontalis* leaf extract-loaded micelles), DMSO (dimethyl sulfoxide, vehicle), EM (empty micelles), Untreated cells (Control)
