# Supplementary material for: Antineoplastic Activity of Podophyllotoxin and Juniper Extracts Encapsulated in MPEG-b-PLA Diblock Copolymer Micelles in Cutaneous Squamous Carcinoma Cells
Source: Int J Mol Sci. 2025 May 28;26(11):5167. doi: 10.3390/ijms26115167 (PMC12154351; doi:10.3390/ijms26115167)
Supplement: Supplementary file 1 [file ijms-26-05167-s001.zip › Suppl Tables 2 to 6 Statistics.pdf]

**Suppl. Table S2.** One-way ANOVA analysis of caspases-3/7 activities in the treated A-431 and HaCaT cells

| Tukey's multiple comparisons test | Mean Diff. | 95.00% CI of diff. | Significance | Adjusted P Value |
|---|---|---|---|---|
| A-431 cells | | | | |
| Co vs. DMSO | 1079 | -1302758 to 1304916 | ns | >0.9999 |
| Co vs. EM | 2071 | -1062508 to 1066650 | ns | >0.9999 |
| Co vs. PPT | -1441661 | -2506240 to -377082 | * | 0.0146 |
| Co vs. nPPT | -1491709 | -2556288 to -427130 | * | 0.0126 |
| Co vs. ETP | -1185470 | -2250049 to -120891 | * | 0.0329 |
| DMSO vs. EM | 992.0 | -1302845 to 1304829 | ns | >0.9999 |
| DMSO vs. PPT | -1442740 | -2746577 to -138903 | * | 0.0337 |
| DMSO vs. nPPT | -1492788 | -2796625 to -188951 | * | 0.0294 |
| DMSO vs. ETP | -1186549 | -2490386 to 117288 | ns | 0.0710 |
| EM vs. PPT | -1443732 | -2508311 to -379153 | * | 0.0145 |
| EM vs. nPPT | -1493780 | -2558359 to -429201 | * | 0.0125 |
| EM vs. ETP | -1187541 | -2252120 to -122962 | * | 0.0326 |
| PPT vs. nPPT | -50048 | -1114627 to 1014531 | ns | >0.9999 |
| PPT vs. ETP | 256191 | -808388 to 1320770 | ns | 0.8909 |
| nPPT vs. ETP | 306239 | -758340 to 1370818 | ns | 0.8099 |
| | | | | |
| HaCaT cells | | | | |
| Co vs. DMSO | 1774 | -256613 to 260161 | ns | >0.9999 |
| Co vs. EM | 1496 | -209476 to 212468 | ns | >0.9999 |
| Co vs. PPT | -809131 | -1020103 to -598159 | *** | 0.0001 |
| Co vs. nPPT | -834549 | -1045521 to -623577 | **** | <0.0001 |
| Co vs. ETP | -542495 | -753467 to -331523 | *** | 0.0008 |
| DMSO vs. EM | -278.0 | -258665 to 258109 | ns | >0.9999 |
| DMSO vs. PPT | -810905 | -1069292 to -552518 | *** | 0.0003 |
| DMSO vs. nPPT | -836323 | -1094710 to -577936 | *** | 0.0003 |
| DMSO vs. ETP | -544269 | -802656 to -285882 | ** | 0.0020 |
| EM vs. PPT | -810627 | -1021599 to -599655 | *** | 0.0001 |
| EM vs. nPPT | -836045 | -1047017 to -625073 | **** | <0.0001 |
| EM vs. ETP | -543991 | -754963 to -333019 | *** | 0.0008 |
| PPT vs. nPPT | -25418 | -236390 to 185554 | ns | 0.9931 |
| PPT vs. ETP | 266636 | 55664 to 477608 | * | 0.0196 |
| nPPT vs. ETP | 292054 | 81082 to 503026 | * | 0.0133 |

Abbreviations: PPT (podophyllotoxin), nPPT (PPT-loaded nanosized micelles), ETP (etoposide), DMSO (dimethyl sulfoxide, vehicle), EM (empty micelles), Co (control, untreated cells); 95% CI – confidence interval

**Suppl. Table S3.** One-way ANOVA analysis of ROS generation in treated A-431 and HaCaT cells

| Tukey's multiple comparisons test | Mean Diff. | 95% CI of diff. | Significant | Summary |
|---|---|---|---|---|
| **A-431 cells** | | | | |
| Co vs. DMSO | -17.00 | -152.8 to 118.8 | No | ns |
| Co vs. EM | -57.00 | -192.8 to 78.76 | No | ns |
| Co vs. PPT | -443.8 | -579.5 to -308.0 | Yes | **** |
| **Co vs. nPPT** | **-195.0** | **-330.8 to -59.24** | **Yes** | ** |
| Co vs. ETP | -982.7 | -1140 to -825.6 | Yes | **** |
| Co vs. $H_2O_2$ | -4850 | -4986 to -4714 | Yes | **** |
| DMSO vs. EM | -40.00 | -175.8 to 95.76 | No | ns |
| DMSO vs. PPT | -426.8 | -562.5 to -291.0 | Yes | **** |
| DMSO vs. nPPT | -178.0 | -313.8 to -42.24 | Yes | ** |
| DMSO vs. ETP | -965.7 | -1123 to -808.6 | Yes | **** |
| DMSO vs. $H_2O_2$ | -4833 | -4969 to -4697 | Yes | **** |
| EM vs. PPT | -386.8 | -522.5 to -251.0 | Yes | **** |
| EM vs. nPPT | -138.0 | -273.8 to -2.235 | Yes | * |
| EM vs. ETP | -925.7 | -1083 to -768.6 | Yes | **** |
| EM vs. $H_2O_2$ | -4793 | -4929 to -4657 | Yes | **** |
| PPT vs. nPPT | 248.8 | 113.0 to 384.5 | Yes | **** |
| PPT vs. ETP | -539.0 | -696.2 to -381.8 | Yes | **** |
| PPT vs. $H_2O_2$ | -4406 | -4542 to -4270 | Yes | **** |
| nPPT vs. ETP | -787.7 | -944.9 to -630.6 | Yes | **** |
| nPPT vs. $H_2O_2$ | -4655 | -4791 to -4519 | Yes | **** |
| ETP vs. $H_2O_2$ | -3867 | -4024 to -3710 | Yes | **** |
| **HaCaT cells** | | | | |
| Co vs. DMSO | -0.5000 | -136.3 to 135.3 | No | ns |
| Co vs. EM | -7.250 | -143.0 to 128.5 | No | ns |
| Co vs. PPT | -150.8 | -286.5 to -14.99 | Yes | * |
| **Co vs. nPPT** | **-44.75** | **-180.5 to 91.01** | **No** | **ns** |
| Co vs. ETP | -426.0 | -583.2 to -268.8 | Yes | **** |
| Co vs. $H_2O_2$ | -4839 | -4974 to -4703 | Yes | **** |
| DMSO vs. EM | -6.750 | -142.5 to 129.0 | No | ns |
| DMSO vs. PPT | -150.3 | -286.0 to -14.49 | Yes | * |
| DMSO vs. nPPT | -44.25 | -180.0 to 91.51 | No | ns |
| DMSO vs. ETP | -425.5 | -582.7 to -268.3 | Yes | **** |
| DMSO vs. $H_2O_2$ | -4838 | -4974 to -4702 | Yes | **** |
| EM vs. PPT | -143.5 | -279.3 to -7.735 | Yes | * |
| EM vs. nPPT | -37.50 | -173.3 to 98.26 | No | ns |
| EM vs. ETP | -418.7 | -575.9 to -261.6 | Yes | **** |
| EM vs. $H_2O_2$ | -4831 | -4967 to -4695 | Yes | **** |
| PPT vs. nPPT | 106.0 | -29.76 to 241.8 | No | ns |
| PPT vs. ETP | -275.3 | -432.4 to -118.1 | Yes | **** |
| PPT vs. $H_2O_2$ | -4688 | -4824 to -4552 | Yes | **** |
| nPPT vs. ETP | -381.2 | -538.4 to -224.1 | Yes | **** |
| nPPT vs. $H_2O_2$ | -4794 | -4930 to -4658 | Yes | **** |
| ETP vs. $H_2O_2$ | -4413 | -4570 to -4255 | Yes | **** |

Abbreviations: PPT (podophyllotoxin), nPPT (PPT-loaded nanosized micelles), ETP (etoposide), DMSO (dimethyl sulfoxide, vehicle), EM (empty micelles), Co (control, untreated cells); 95% CI – confidence interval

**Suppl. Table S4.** Comparison of cell percentage in the different phases of the cell cycle within each of the treated A-431 and HaCaT cell lines (FACS analysis) – One-way ANOVA.

| Tukey's multiple comparisons test | Mean Diff. | 95% CI of diff. | Significance | Adjusted P Value |
|---|---|---|---|---|
| HaCaT, 40h | | | | |
| Control vs. DMSO | 0.003900 | -0.04480 to 0.05260 | ns | 0.9992 |
| Control vs. EM | -0.003400 | -0.05210 to 0.04530 | ns | 0.9995 |
| Control vs. PPT | -0.2269 | -0.2756 to -0.1782 | **** | < 0.0001 |
| Control vs. nPPT | -0.1501 | -0.1988 to -0.1014 | **** | < 0.0001 |
| DMSO vs. EM | -0.007300 | -0.05600 to 0.04140 | ns | 0.9910 |
| DMSO vs. PPT | -0.2308 | -0.2795 to -0.1821 | **** | < 0.0001 |
| DMSO vs. nPPT | -0.1540 | -0.2027 to -0.1053 | **** | < 0.0001 |
| EM vs. PPT | -0.2235 | -0.2722 to -0.1748 | **** | < 0.0001 |
| EM vs. nPPT | -0.1467 | -0.1954 to -0.09800 | **** | < 0.0001 |
| PPT vs. nPPT | 0.07677 | 0.02806 to 0.1255 | ** | 0.0011 |
| A-431, 40h | | | | |
| Control vs. DMSO | -0.01195 | -0.05413 to 0.03023 | ns | 0.9122 |
| Control vs. EM | -0.007875 | -0.05005 to 0.03430 | ns | 0.9795 |
| Control vs. PPT | -0.1710 | -0.2132 to -0.1288 | **** | < 0.0001 |
| Control vs. nPPT | -0.006575 | -0.04875 to 0.03560 | ns | 0.9895 |
| DMSO vs. EM | 0.004075 | -0.03810 to 0.04625 | ns | 0.9983 |
| DMSO vs. PPT | -0.1590 | -0.2012 to -0.1168 | **** | < 0.0001 |
| DMSO vs. nPPT | 0.005375 | -0.03680 to 0.04755 | ns | 0.9951 |
| EM vs. PPT | -0.1631 | -0.2053 to -0.1209 | **** | < 0.0001 |
| EM vs. nPPT | 0.0013 | -0.04088 to 0.04348 | ns | > 0.9999 |
| PPT vs. nPPT | 0.1644 | 0.1222 to 0.2066 | **** | < 0.0001 |

Abbreviations: PPT (podophyllotoxin), nPPT (PPT-loaded nanosized micelles), ETP (etoposide), DMSO (dimethyl sulfoxide, vehicle), EM (empty micelles), Co (control, untreated cells); 95% CI – confidence interval

**Suppl. Table S5.** Comparison of the cell percentage in different phases of the cell cycle between treated HaCaT and A-431 cells (FACS analysis) – Two-way ANOVA.

| Sidak's multiple comparisons test | Mean Diff. | 95% CI of diff. | Significance | Adjusted P Value |
|---|---|---|---|---|
| A-431, 40h vs. HaCaT, 40h | | | | |
| Control G1 | 2.923 | -4.560 to 10.41 | ns | 0.9832 |
| Control S | -0.9367 | -8.420 to 6.547 | ns | > 0.9999 |
| Control G2 | -0.3700 | -7.853 to 7.113 | ns | > 0.9999 |
| DMSO G1 | 2.523 | -4.960 to 10.01 | ns | 0.9960 |
| DMSO S | -3.720 | -11.20 to 3.763 | ns | 0.8862 |
| DMSO G2 | 0.8800 | -6.603 to 8.363 | ns | > 0.9999 |
| EM G1 | 7.117 | -0.3668 to 14.60 | ns | 0.0754 |
| EM S | -8.560 | -16.04 to -1.077 | * | 0.0137 |
| EM G2 | 0.2633 | -7.220 to 7.747 | ns | > 0.9999 |
| PPT G1 | 2.170 | -5.313 to 9.653 | ns | 0.9992 |
| PPT S | 2.047 | -5.437 to 9.530 | ns | 0.9996 |
| PPT G2 | -3.187 | -10.67 to 4.297 | ns | 0.9642 |
| nPPT G1 | 21.90 | 14.42 to 29.38 | **** | < 0.0001 |
| nPPT S | -10.88 | -18.36 to -3.397 | *** | 0.0006 |
| nPPT G2 | -7.820 | -15.30 to -0.3365 | * | 0.0338 |

Abbreviations: PPT (podophyllotoxin), nPPT (PPT-loaded nanosized micelles), ETP (etoposide), DMSO (dimethyl sulfoxide, vehicle), EM (empty micelles), Co (control, untreated cells); 95% CI – confidence interval

**Suppl. Table S6.** Comparison - G2/M vs. G0/G1 phase ratio in treated A-431 and HaCaT cells (FACS analysis) – Two-way ANOVA.

| Sidak's multiple comparisons test | Mean Diff. | 95% CI of diff. | Significance | Adjusted P Value |
|---|---|---|---|---|
| A-431, 40h vs. HaCaT, 40h | | | | |
| Control | -0.005708 | -0.04690 to 0.03549 | ns | 0.9977 |
| DMSO | 0.01014 | -0.03105 to 0.05134 | ns | 0.9688 |
| EM | -0.001233 | -0.04243 to 0.03996 | ns | > 0.9999 |
| PPT | -0.0616 | -0.1028 to -0.02040 | ** | 0.0017 |
| nPPT | -0.1492 | -0.1904 to -0.1080 | **** | < 0.0001 |

Abbreviations: PPT (podophyllotoxin), nPPT (PPT-loaded nanosized micelles), ETP (etoposide), DMSO (dimethyl sulfoxide, vehicle), EM (empty micelles), Co (control, untreated cells); 95% CI – confidence interval
